# Coumarin-chalcone hybrid LM-021 and indole derivative NC009-1 targeting inflammation and oxidative stress to protect BE(2)-M17 cells against α-synuclein toxicity

**DOI:** 10.18632/aging.204954

**Published:** 2023-08-11

**Authors:** Pei-Ning Yang, Wan-Ling Chen, Jun-Wei Lee, Chih-Hsin Lin, Yi-Ru Chen, Chung-Yin Lin, Wenwei Lin, Ching-Fa Yao, Yih-Ru Wu, Kuo-Hsuan Chang, Chiung-Mei Chen, Guey-Jen Lee-Chen

**Affiliations:** 1 Department of Life Science, National Taiwan Normal University, Taipei 11677, Taiwan; 2 Department of Neurology, Chang Gung Memorial Hospital, Chang Gung University College of Medicine, Taoyuan 33302, Taiwan; 3 Department of Chemistry, National Taiwan Normal University, Taipei 11677, Taiwan; 4 Medical Imaging Research Center, Institute for Radiological Research, Chang Gung University/Chang Gung Memorial Hospital, Taoyuan 33302, Taiwan

**Keywords:** Parkinson’s disease/α-synuclein, NLRP1/3, IL-1β/IL-6 signaling, GSH/GSSG, therapeutics

## Abstract

Parkinson’s disease (PD) is featured mainly by the loss of dopaminergic neurons and the presence of α-synuclein-containing aggregates in the substantia nigra of brain. The α-synuclein fibrils and aggregates lead to increased oxidative stress and neural toxicity in PD. Chronic inflammation mediated by microglia is one of the hallmarks of PD pathophysiology. In this report, we showed that coumarin-chalcone hybrid LM-021 and indole derivative NC009-1 reduced the expression of major histocompatibility complex-II, NLR family pyrin domain containing (NLRP) 3, caspase-1, inducible nitric oxide synthase, interleukin (IL)-1β, IL-6, and tumor necrosis factor (TNF)-α in α-synuclein-activated mouse BV-2 microglia. Release of pro-inflammatory mediators including nitric oxide, IL-1β, IL-6 and TNF-α was also mitigated. In BE(2)-M17 cells expressing A53T α-synuclein aggregates, LM-021 and NC009-1 reduced α-synuclein aggregation, neuroinflammation, oxidative stress and apoptosis, and promoted neurite outgrowth. These protective effects were mediated by downregulating NLRP1, IL-1β and IL-6, and their downstream pathways including nuclear factor (NF)-κB inhibitor alpha (IκBα)/NF-κB P65 subunit (P65), c-Jun N-terminal kinase (JNK)/proto-oncogene c-Jun (JUN), mitogen-activated protein kinase 14 (P38)/signal transducer and activator of transcription (STAT) 1, and Janus kinase 2 (JAK2)/STAT3. The study results indicate LM-021 and NC009-1 as potential new drug candidates for PD.

## INTRODUCTION

Parkinson’s disease (PD), a common neurodegenerative disorder affecting the elderly, has clinical features of resting tremor, rigidity, bradykinesia and postural instability. These symptoms result predominantly from a massive loss of dopaminergic neurons in the substantia nigra and subsequent depletion of dopamine in their projections. The pathological hallmark of PD is the presence of α-synuclein-containing Lewy bodies and Lewy neuritis in the subcortical regions of the brain [1]. Duplication, triplication and point mutations of α-synuclein (SNCA) gene cause conformation-changed α-synuclein that tends to form aggregates to cause detrimental effects on neurons [2]. Several pathways have been attributed to neurodegeneration, including abnormal loads of misfolded proteins, mitochondrial abnormalities, increased oxidative stress and inflammation, ubiquitin-proteasome dysfunction, impaired autophagy-lysosome and mitophagy, deficient synaptic exocytosis and endocytosis, and defective endosomal trafficking [3]. Despite the disease etiology remaining to be clarified, substantial evidence has shown that oxidative stress and neuroinflammation play an important role in the pathophysiology of PD [4, 5]. Therefore, strategies or agents that reduce oxidative stress and inflammation may serve as potential candidates for PD treatment.

Lines of evidence have demonstrated that microglial activation, nuclear factor kappa B (NF-κB)-induced neuroinflammation, and secretion of inflammatory factors may significantly contribute to the neurodegeneration of PD [6, 7]. Aggregated α-synuclein could activate microglia to release proinflammatory cytokines, interleukin (IL)-1β and IL-6, which leads to disease progression of PD [8–10]. Neuroinflammation is also likely to mediate transmission of misfolded α-synuclein in PD [11].

Nucleotide-binding oligomerization domain-like receptors (NLRs) are intracellular receptors which play key roles in innate immunity and inflammation. Upon sensing danger signals such as presence of infection, noxious substances or metabolic perturbations, NLRs induce the assembly of large complexes composed of NLR family pyrin domain containing (NLRP) sensor, ASC adaptor with N-terminal NLRP sensor-interaction PYD domain and C-terminal caspase-recruitment CARD domain, and the caspase-1 (CASP1) protease [12]. CASP1 activation results in the processing and secretion of IL-1β and IL-18. Both oxidative stress and insoluble α-synuclein aggregates can trigger the inflammasome pathway in PD brains [13]. Aggregated α-synuclein can be up-taken by microglia, which activates the microglial NLR family pyrin domain containing 3 (NLRP3) inflammasome and causes the generation of mitochondrial reactive oxygen species (ROS) [14], and compounds suppressing NLRP3 inflammasome activation show neuroprotection on *in vivo* and *in vitro* PD models [15–19]. While NLRP3 inflammasome is mainly produced in reactive microglia, NLR family pyrin domain containing 1 (NLRP1) is majorly generated by neurons and neuronal NLRP1 inflammasome activation regulates inflammatory IL-1β production and axonal degeneration [20].

Previously, we have developed two in-house compounds, the coumarin–chalcone derivative LM-021 [21] and the indole derivative compound NC009-1 [22–24] and shown their protective effects on tauopathy and Aβ cell models, and/or spinocerebellar ataxia type 17 cell and mouse models. Whether these compounds have anti-inflammatory effects is not known. We proposed that α-synuclein induces microglial activation and release of IL-1β, IL-6 and tumor necrosis factor (TNF)-α to activate NLRP1 in neuronal cells, which leads to elevated oxidative stress and neuronal damage. In the present study, we first examined the blood–brain barrier (BBB) permeability, 1,1-diphenyl-2-picrylhydrazyl (DPPH) free radical scavenging activity, antioxidant capacity, and cytotoxicity of LM-021 and NC009-1. Second, we examined the anti-aggregatory effects of LM-021 and NC009-1 by using thioflavin T binding assay. Third, we examined the anti-inflammatory effects of LM-021 and NC009-1 on α-synuclein-activated BV-2 microglia and NLRP3 inflammasome pathway. Fourth, we tested the neuroprotective effects of LM-021 and NC009-1 on α-synuclein-inflamed BE(2)-M17 cells expressing A53T α-synuclein (SNCA)-GFP. Finally, we investigated if these two compounds mitigate the NLRP1, CASP1, IL-1β, IL-6, TNF-α, and their downstream pathways to provide protective effects.

## RESULTS

### Tested compounds, radical scavenging and cytotoxicity

**Figure 1:**
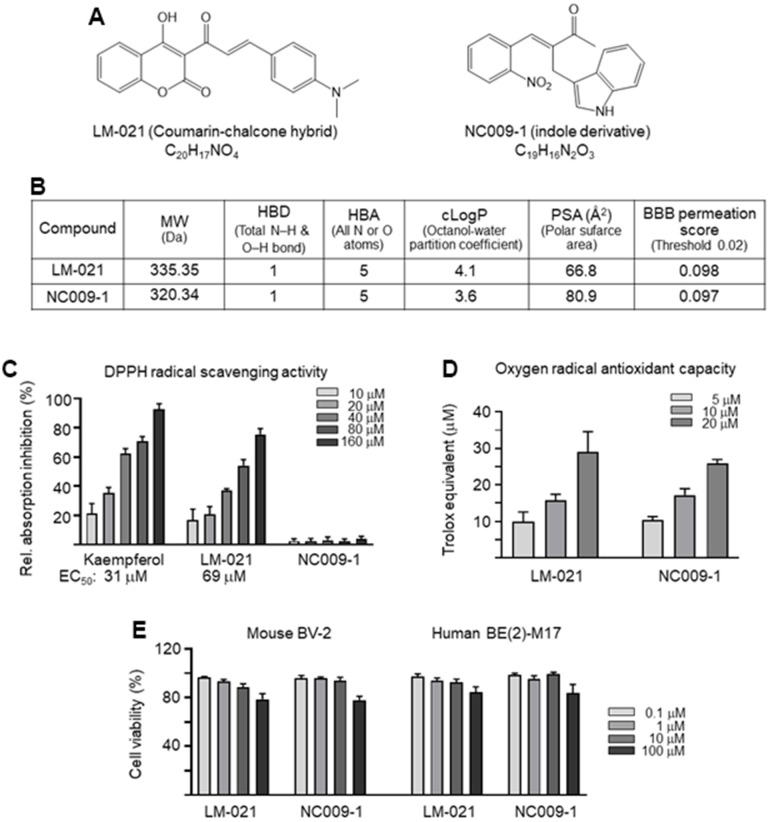
**LM-021 and NC009-1.** (**A**) Structure and formula of LM-021 and NC009-1. (**B**) Molecular weight (MW), hydrogen bond donor (HBD), hydrogen bond acceptor (HBA), calculated partition coefficient of octanol/water (cLogP), polar surface area (PSA), and blood–brain barrier (BBB) permeation score of LM-021 and NC009-1. (**C**) DPPH free radical scavenging activity of kaempferol (as a positive control), LM-021 and NC009-1 (10−160 μM) (n = 3). Shown below are the EC_50_ values. (**D**) Trolox equivalent oxygen radical antioxidant capacity of LM-021 and NC009-1 (5−20 μM) (n = 3). (**E**) Cytotoxicity of LM-021 and NC009-1 against mouse BV-2 and human BE(2)-M17 cells determined by using the MTT assay. Cells were treated with each test compound (0.1–100 μM) and cell viability was analyzed the next day (n = 3). For normalization, the relative viability of untreated cells was set at 100%.

Two synthetic compounds, coumarin-chalcone hybrid LM-021 and indole derivative NC009-1 (Figure 1A), were examined. Oral bioavailability was predicted based on molecular weight (MW, 335.35 and 320.34), numbers of hydrogen-bond donor (HBD, 1) and acceptor (HBA, 5), and calculated partition coefficient of octanol/water (cLogP, 4.1 and 3.6) (Figure 1B). According to the calculated polar surface area (PSA) (66.8 Å^2^ and 80.9 Å^2^) and predicted BBB permeation score (0.098 and 0.097), both compounds displayed potential of BBB penetration (Figure 1B). The bioavailability of LM-021 and NC009-1 is intimately related to the efficiency of their BBB penetration.

To assess the antioxidative properties of LM-021 and NC009-1, DPPH radical scavenging activity and oxygen radical antioxidant capacity were examined. LM-021 had EC_50_ value of 69 μM, while NC009-1 displayed no DPPH radical scavenging activity, (Figure 1C). In addition, oxygen radical antioxidant capacity was determined based on the trolox standard curve. LM-021 and NC009-1 at 5−20 μM concentration displayed similar trolox equivalent activity (LM-021: 10−29 μM, NC009-1: 10−26 μM) (Figure 1D). The cytotoxicity of LM-021 and NC009-1 in mouse BV-2 microglia and human BE(2)-M17 cells was examined by using MTT assay. Both compounds demonstrated cell viability of 79–78% and 85–84%, respectively in 100 μM compound-treated BV-2 and BE(2)-M17 cells (Figure 1E).

### α-Synuclein amyloid fibrils and examination

**Figure 2:**
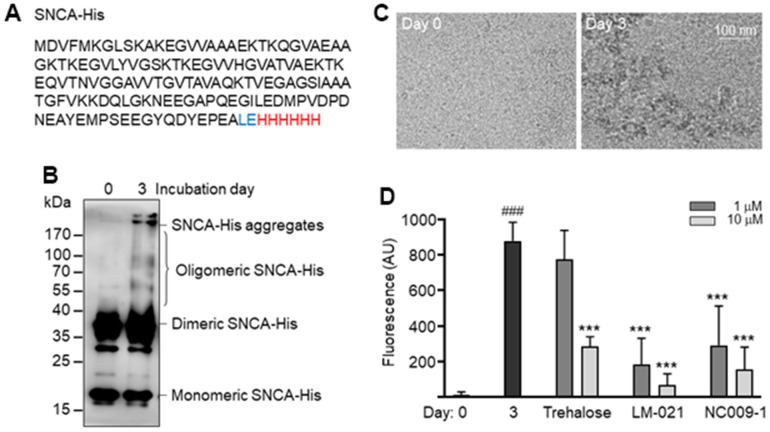
**SNCA-His protein and aggregation.** (**A**) SNCA-His protein. α-Synuclein (140 amino acids, marked in black) was in-frame fused to a 6-amino acid His tag (marked in red) via a 2 amino-acid linker (Leu and Glu, marked in blue). (**B**) SNCA-His aggregates formation. SNCA-His (20 μM in 300 μl buffer) was incubated at 37° C for 3 days and aggregates were examined by 10% SDS-PAGE gel and immunoblot probing with the α-synuclein antibody. (**C**) Cryo-TEM structure of aggregated SNCA-His. (**D**) Thioflavin T binding assay for SNCA-His aggregation. SNCA-His protein (20 μM) was incubated with trehalose, LM-021 or NC009-1 (1−10 μM) at 37° C for three days, and aggregation was analyzed by measuring thioflavin T fluorescence intensity (n = 3). *P* values: day 0 vs. day 3 (^###^: *P* < 0.001), or with vs. without compound treatment (***: *P* < 0.001).

α-Synuclein is an intrinsically destabilized protein that tends to form β-sheet-rich amyloid fibrils. His-tagged recombinant α-synuclein (SNCA-His) protein (Figure 2A) was produced in *E. coli*. After incubation of 20 μM soluble SNCA-His at 37° C for 3 days, oligomeric and aggregated SNCA-His were readily seen by immunoblot probing with the α-synuclein antibody (Figure 2B), which is in agreement with a distinguishing morphology of fibrils under cryogenic transmission electron microscopy (cryo-TEM) (Figure 2C). By quantifying thioflavin T fluorescence, increased SNCA-His aggregation was also observed after 3 days of incubation at 37° C (from 14 arbitrary unit (AU) to 880 AU; *P* < 0.001). To assess the chemical chaperone activity in assisting α-synuclein folding, LM-021 or NC009-1 was included during the incubation period. As Figure 2D showed, SNCA-His aggregation was significantly reduced by LM-021 at 1–10 μM (184–68 AU; *P* < 0.001) and NC009-1 at 1–10 μM (291–154 AU; *P* < 0.001).

### Anti-inflammatory potentials of LM-021 and NC009-1 in α-synuclein induced microglial activation

**Figure 3:**
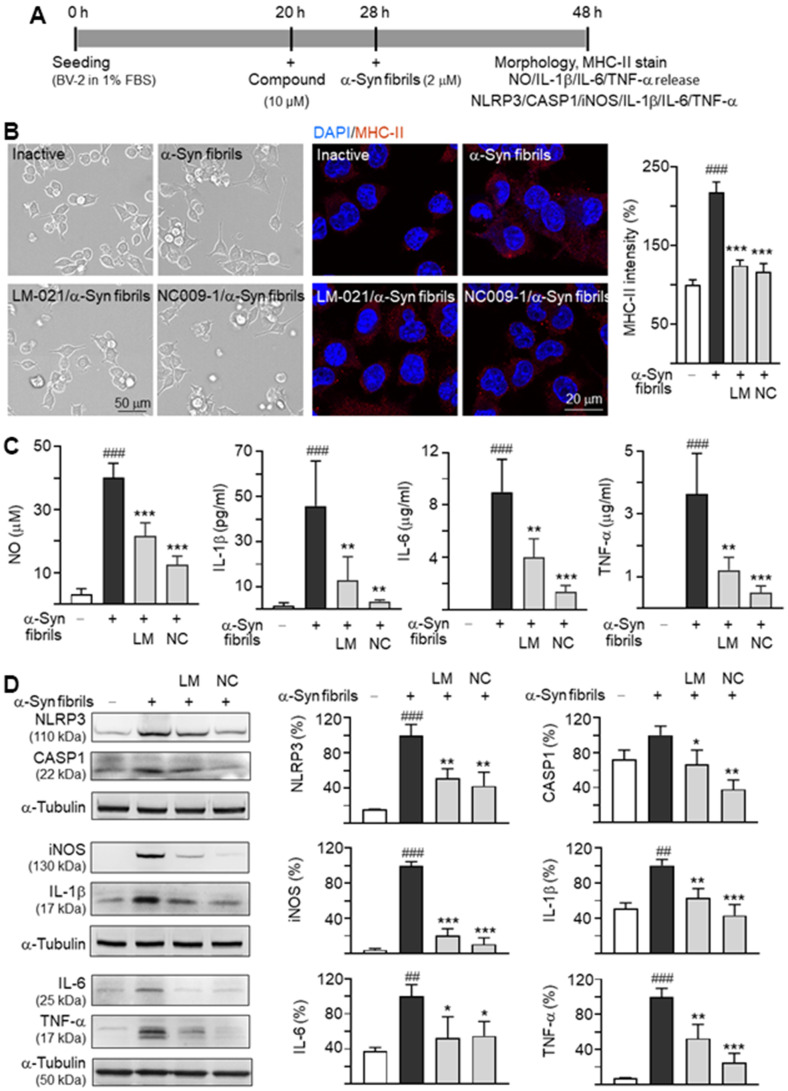
**Inflammation in BV-2 microglia induced by α-synuclein.** (**A**) Experimental flow chart. BV-2 microglia were plated in 1% FBS on day 1. After 20 h, the cells were pretreated with the test compound (10 μM) for 8 h, and then treated with α-synuclein fibrils (2 μM). 20 h later, the BV-2 cells were analyzed for (**B**) morphology, MHC-II expression, (**C**) NO/IL-1β/IL-6/TNF-α release into culture medium, and (**D**) cellular NLRP3/CASP1/iNOS/IL-1β/IL-6/TNF-α expression (n = 3). For normalization, the relative MHC-II level in inactive cells or NLRP3/CASP1/iNOS/IL-1β/IL-6/TNF-α level in activated cells was set as 100%. *P* values: inactivated vs. activated cells (^##^: *P* < 0.01, ^###^: *P* < 0.001), or with vs. without treatment of compound (*: *P* < 0.05, **: *P* < 0.01, ***: *P* < 0.001).

The inflammation triggered by oxidative stress is one of the causes of many neurodegenerative diseases. To examine anti-inflammatory potentials of LM-021 and NC009-1, mouse BV-2 cells in 1% FBS were pre-treated with each of the test compounds (10 μM) for 8 h, followed by adding with α-synuclein fibrils (2 μM) for 20 h (Figure 3A). As shown in Figure 3B, α-synuclein fibrils treatment transformed the resting ramified BV-2 into an elongated morphology with extended processes, accompanied with up-regulation of anti-major histocompatibility complex (MHC)-II (218%; *P* < 0.001). However, pre-treatment with LM-021 or NC009-1 counteracted the altered morphology and the elevated MHC-II expression (124–117%; *P* < 0.001). Significantly increased production of nitric oxide (NO) (from 3.1 μM to 40.1 μM), IL-1β (from 1.6 pg/ml to 45.7 pg/ml), IL-6 (from 0 μg/ml to 9.0 μg/ml) and TNF-α (from 0.1 μg/ml to 3.6 μg/ml) in culture medium was also observed (*P* < 0.001) after α-synuclein fibrils stimulation. LM-021 pre-treatment significantly reduced the levels of NO (21.7 μM; *P* < 0.001), IL-1β (12.9 pg/ml; *P* = 0.007), IL-6 (4.0 μg/ml; *P* = 0.002) and TNF-α (1.2 μg/ml; *P* = 0.002) (Figure 3C). This is also true for NC009-1 pre-treatment: NO (12.5 μM; *P* < 0.001), IL-1β (3.6 pg/ml; *P* = 0.001), IL-6 (1.4 μg/ml; *P* < 0.001) and TNF-α (0.5 μg/ml; *P* < 0.001) (Figure 3C).

NLRP3 inflammasome mediates caspase-1 activation and release of IL-1β from microglia. The protein levels of NLRP3, CASP1, inducible nitric oxide synthase (iNOS), IL-1β, IL-6 and TNF-α with and without α-synuclein fibrils and/or compound treatment were then examined in α-synuclein-stimulated mouse BV-2 cells using Western blot. As shown in Figure 3D, α-synuclein fibrils addition increased the expressions of NLRP3 (from 15% to 100%; *P* < 0.001), iNOS (from 4% to 100%; *P* < 0.001), IL-1β (from 51% to 100%; *P* = 0.001), IL-6 (from 37% to 100%; *P* = 0.006) and TNF-α (from 7% to 100%; *P* < 0.001). For CASP1, the increase (from 73% to 100%) was minimal and not statistically significant. Treatment with LM-021 at 10 μM concentration significantly reduced the levels of NLRP3 (51%), CASP1 (67%), iNOS (21%), IL-1β (63%), IL-6 (53%) and TNF-α (53%) (*P* = 0.044–<0.001) involved in inflammasome and neuroinflammation pathways. Treatment with NC009-1 at 10 μM concentration also significantly reduced the levels of these inflammatory markers: NLRP3 (42%), CASP1 (38%), iNOS (11%), IL-1β (43%), IL-6 (55%), TNF-α (25%) (*P* = 0.037–<0.001).

### Neuroprotective effects of LM-021 and NC009-1 on A53T SNCA-GFP BE(2)-M17 cells

Human neuroblastoma BE(2)-M17 cells with induced expression of green fluorescent protein-tagged wild type (WT) or A53T α-synuclein (SNCA-GFP) were generated and blasticidin-resistance clones WT-4, WT-5, A53T-8 and A53T-11 were selected, expanded, and differentiated with retinoic acid (5 μM) (Supplementary Figure 1). After inducing α-synuclein expression in the presence or absence of preformed α-synuclein fibrils (0.1 μM) for five days, ProteoStat®-stained α-synuclein aggregation and neurite outgrowth were characterized for these clones (Supplementary Figure 2).

**Figure 4:**
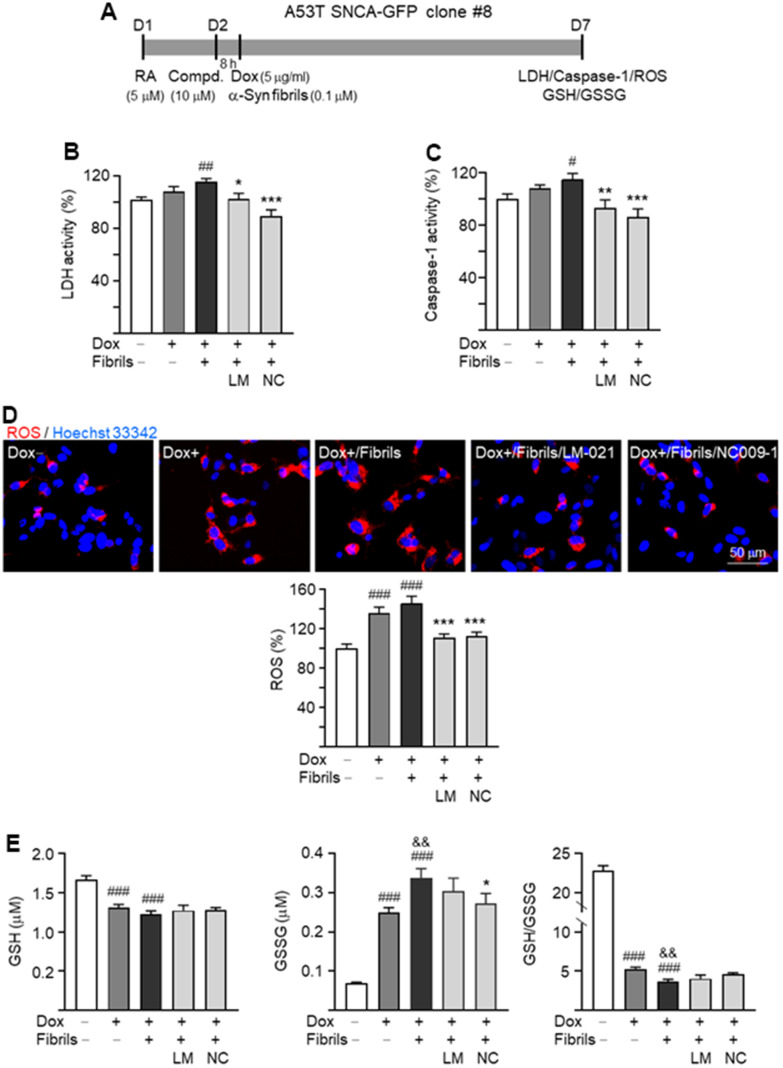
**Neuroprotective effects of LM-021 and NC009-1 on A53T-8 SNCA-GFP BE(2)-M17 cells.** (**A**) Experimental flow chart. Cells were plated with the presence of retinoic acid (RA, 5 μM) on day 1. On day 2, cells were treated with LM-021 or NC009-1 (10 μM) for 8 h, followed by inducing SNCA-GFP expression (Dox, 5 μg/ml), and further added with α-synuclein fibril (0.1 μM). On day 7, cellular LDH, caspase-1, ROS and GSH/GSSG were assessed. (**B**) LDH activity, (**C**) caspase-1 activity, (**D**) ROS production and (**E**) GSH/GSSG ratio of A53T-8 SNCA-GFP BE(2)-M17 cells with or without induced SNCA-GFP expression, preformed fibril addition, or LM-021/NC009-1 treatment (n = 3). *P* values: with vs. without doxycycline induction (^#^: *P* < 0.05, ^##^: *P* < 0.01, ^###^: *P* < 0.001), with vs. without addition of fibrils (^&&^: *P* < 0.01), or with vs. without compound treatment (*: *P* < 0.05, **: *P* < 0.01, ***: *P* < 0.001).

To evaluate neuroprotective effects of LM-021 and NC009-1 in A53T SNCA-GFP BE(2)-M17 cells, lactate dehydrogenase (LDH) release, caspase-1 activity and ROS production in A53T-8 cells were examined (Figure 4A). As shown in Figure 4B, 4C, A53T α-synuclein overexpression plus preformed α-synuclein fibrils addition increased LDH release (116%, *P* = 0.008) and caspase-1 (115%, *P* = 0.020) activity, and the tested compound treatments reduced LDH to 103% (LM-021; *P* = 0.011) or 89% (NC009-1; *P* < 0.001) and reduced caspase-1 to 93% (LM-021; *P* = 0.002) or 86% (NC009-1; *P* < 0.001) in A53T-8 cells. In addition, A53T α-synuclein overexpression plus preformed α-synuclein fibrils addition increased cellular ROS (146%; *P* < 0.001), and the tested compound treatments reduced ROS to 113% (LM-021) or 111% (NC009-1) (*P* < 0.001) (Figure 4D). Similar changes of LDH, caspase-1 and ROS were also observed after treatment with LM-021 or NC009-1 in A53T-11 cells (Supplementary Figure 3).

Moreover, cellular levels of reduced (GSH) and oxidized (GSSG) glutathione and GSH/GSSG ratio in A53T-8 were assessed. A53T α-synuclein overexpression plus addition of preformed α-synuclein fibrils decreased GSH (from 1.67 μM to 1.23 μM; *P* < 0.001), increased GSSG (from 0.07 μM to 0.34 μM; *P* < 0.001), and reduced GSH/GSSG ratio (from 22.8 to 3.7; *P* < 0.001), whereas only reduction of GSSG was observed (from 0.34 μM to 0.27 μM; *P* = 0.037) with NC009-1 treatment (Figure 4E).

**Figure 5:**
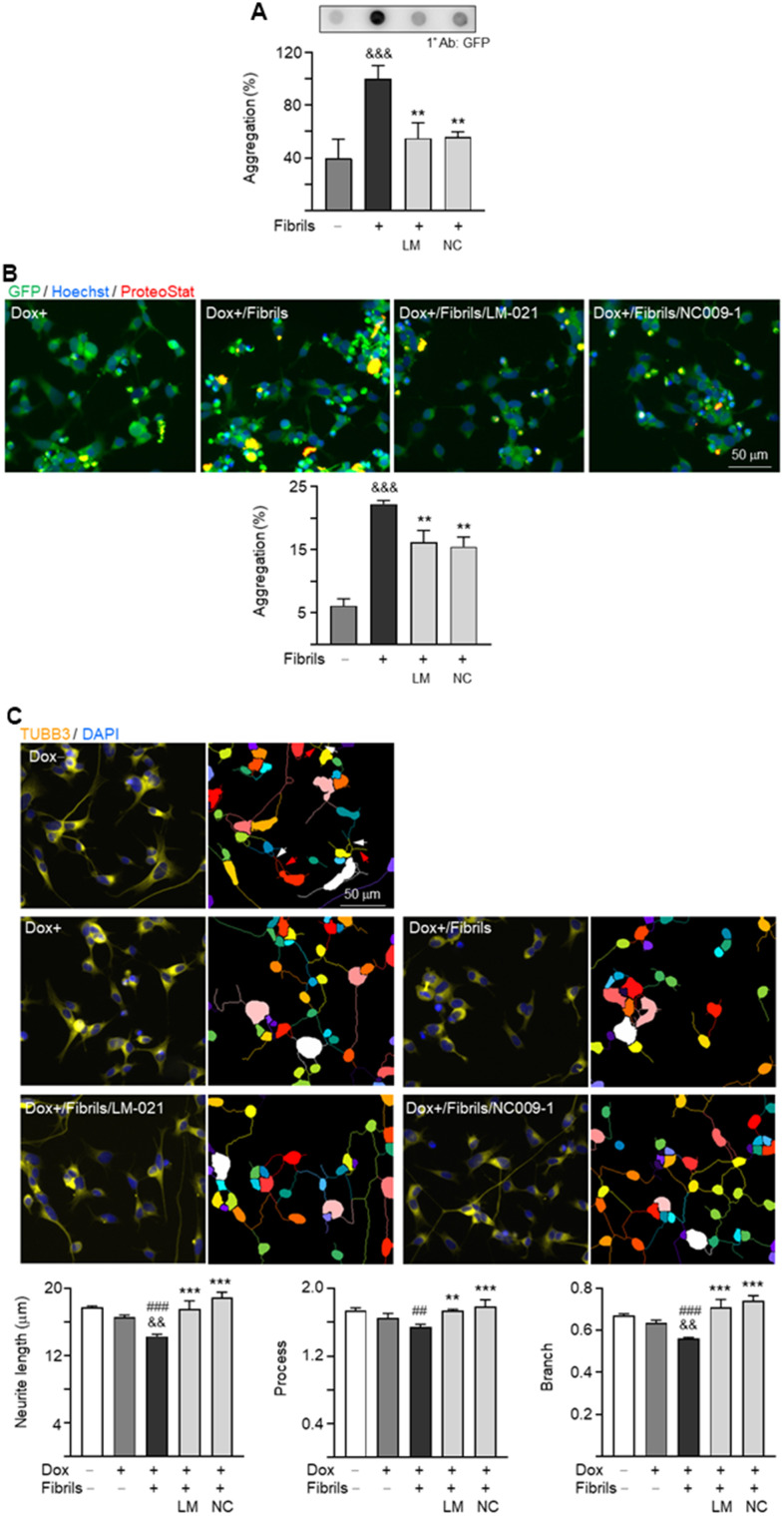
**α-Synuclein aggregation reduction and neurite outgrowth promotion of LM-021 and NC009-1 on A53T-8 SNCA-GFP BE(2)-M17 cells.** The cells were added with retinoic acid on day 1, and treated with compound, doxycycline and α-synuclein fibril on day 2. On day 7, α-synuclein aggregation and neurite outgrowth were assessed. (**A**) α-Synuclein aggregation analyzed by filter trap assay detected by using a GFP antibody (n = 3). To normalize, the α-synuclein aggregates with fibril addition was set as 100%. (**B**) Fluorescent microscopy images of SNCA-GFP-expressing cells (green) with or without preformed fibril addition or compound treatment and quantification of percentage of aggregated cells (n = 3). Nuclei were detected with Hoechst 33342 (blue) and overlapped SNCA-GFP and ProteoStat®-stained (red) aggregates marked (yellow). (**C**) Fluorescent microscopy images of SNCA-GFP cells stained with TUBB3 for quantifying neurite outgrowth with or without preformed fibril addition or compound treatment, with nuclei detected (DAPI, blue). Also shown were images of the neurites and cell bodies being outlined with different colors to indicate different neurons for quantifying neurite outgrowth. In uninduced cells, processes and branches are indicated with red and white arrows, respectively. Quantification of neurite length, brunch and process was shown below (n = 3). *P* values: with vs. without doxycycline addition (^##^: *P* < 0.01, ^###^: *P* < 0.001), with vs. without fibrils addition (^&&^: *P* < 0.01, ^&&&^: *P* < 0.001), or with vs. without compound treatment (**: *P* < 0.01, ***: *P* < 0.001).

The effects of LM-021 and NC009-1 on α-synuclein aggregation in these A53T-8 cells were assessed by filter trap assay and ProteoStat® stain. Treatment of LM-021 or NC009-1 led to significant aggregation reduction: from 100% to 55% (LM-021) or 56% (NC009-1) (*P* = 0.005–0.004) by filter trap assay (Figure 5A). Quantification of percentage of aggregated cells also revealed 6–7% reduction of aggregated cells (from 22% to 16–15%; *P* = 0.003–0.002) (Figure 5B). In consistent with α-synuclein aggregation, the tested compound treatments significantly promoted neurite outgrowth in A53T-8 SNCA-GFP BE(2)-M17 cells, length: from 14.3 μm to 17.5 μm (LM-021) or 18.9 μm (NC009-1), process: from 1.55 to 1.74 (LM-021) or 1.79 (NC009-1), branch: from 0.56 to 0.71 (LM-021) or 0.74 (NC009-1) (*P* = 0.004–<0.001) (Figure 5C). Similar changes of α-synuclein aggregation and neurite outgrowth were also observed after treatment with LM-021 or NC009-1 in A53T-11 cells (Supplementary Figure 4).

### Down-regulation of NLRP1 inflammasome pathway and up-regulation of cellular redox signaling by LM-021 and NC009-1 in SNCA-GFP BE(2)-M17 cells

**Figure 6:**
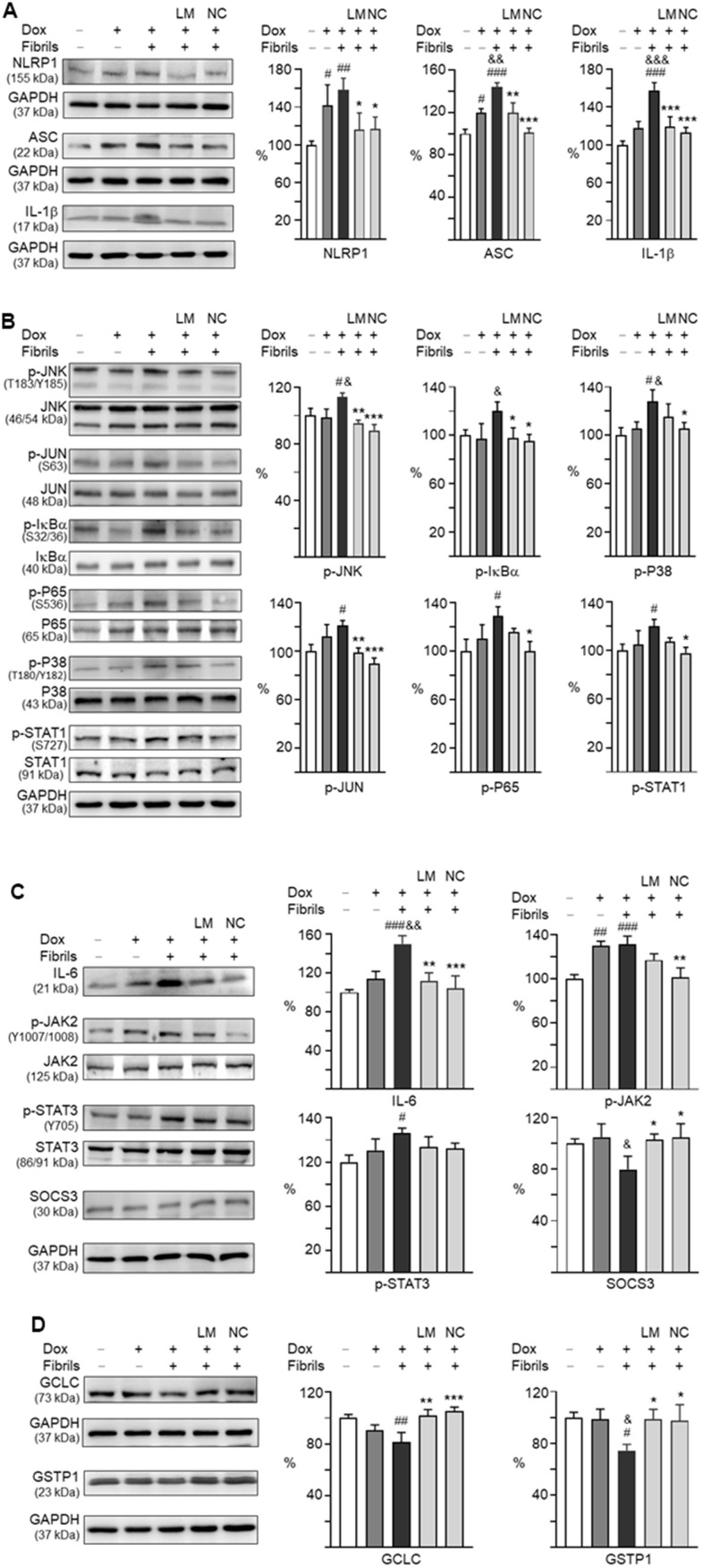
**Effects of LM-021 and NC009-1 on IL-1β- and IL-6-mediated inflammatory and GCLC- and GSTP1-mediated redox signaling in A53T-8 SNCA-GFP BE(2)-M17 cells.** Western blot analysis of (**A**) NLRP1, NLRP3, ASC, IL-1β, (**B**) JNK (T183/Y185), JUN (S63), IκBα (S32/36), P65 (S536), P38 (T180/Y182), STAT1 (S727), (**C**) IL-6, JAK2 (Y1007/1008), STAT3 (Y705), and SOCS3, and (**D**) GCLC and GSTP1. GAPDH was used as an internal control (n = 3). For normalization, the NLRP1, NLRP3, ASC, IL-1β, JNK, JUN, IκBα, P65, P38, STAT1, IL-6, JAK2, STAT3, SOCS3, GCLC and GSTP1 in uninduced cells was set at 100%. *P* values: with vs. without doxycycline induction (^#^: *P* < 0.05, ^##^: *P* < 0.01 and ^###^: *P* < 0.001), with vs. without fibril addition (^&^: *P* < 0.05, ^&&^: *P* < 0.01 and ^&&&^: *P* < 0.001), or with vs. without compound treatment (*: *P* < 0.05, **: *P* < 0.01 and ***: *P* < 0.001).

Neuronal NLRP1 inflammasome activation regulates inflammatory IL-1β production and axonal degeneration. We thus examined the expression of NLRP1, PYD and CARD domain containing ASC, as well as IL-1β in α-synuclein-stimulated neuroinflammation of A53T-8 SNCA-GFP BE(2)-M17 cells. As shown in Figure 6A, induced expression of A53T α-synuclein in BE(2)-M17 cells augmented the expressions of NLRP1 (142%, *P* = 0.038), ASC (120%, *P* = 0.010), and IL-1β (118%, *P* = 0.094), although the increase of IL-1β was not statistically significant. These up-regulations were further exaggerated by adding preformed α-synuclein fibrils compared to uninduced cells (NLRP1: 158%, *P* = 0.005; ASC: 144%, *P* < 0.001; IL-1β: 158%, *P* < 0.001). LM-021 treatment reduced the levels of NLRP1 (116%, *P* = 0.040), ASC (120%, *P* = 0.002) and IL-1β (119%, *P* < 0.001). NC009-1 treatment also reduced the levels of NLRP1 (117%, *P* = 0.044), ASC (101%, *P* < 0.001) and IL-1β (113%, *P* < 0.001).

Upon binding to its receptor, IL-1β activates MAPK/P38 and MAPK/JNK pathways to participate in inflammatory cell signaling [25]. In addition, IL-1 could induce NF-κB activity [26]. Therefore, we examined the expression of these signaling pathways in α-synuclein-stimulated neuroinflammation of A53T SNCA-GFP-expressing BE(2)-M17 cells by Western blotting. As shown in Figure 6B, p-JNK (113%, *P* = 0.029), p-JUN (121%, *P* = 0.012), p-P65 (131%, *P* = 0.011), p-P38 (128%, *P* = 0.010) and p-STAT1 (120%, *P* = 0.025) levels were elevated with induced α-synuclein expression plus α-synuclein fibrils addition. In cells expressing α-synuclein, addition of α-synuclein fibrils increased p-IκBα level (120%, *P* = 0.042). Treatment with LM-021 reduced the p-JNK (95%, *P* = 0.003), p-JUN (98%, *P* = 0.008) and p-IκBα (98%, *P* = 0.048) levels. In addition to p-JNK (90%, *P* < 0.001), p-JUN (89%, *P* < 0.001) and p-IκBα (95%, *P* = 0.027), treatment with NC009-1 also reduced the p-P65 (100%, *P* = 0.011), p-P38 (106%, *P* = 0.038) and p-STAT1 (98%, *P* = 0.014) levels. There were no significant changes in total JNK (106–101%), JUN (107–105%), IκBα (110–106%), P65 (108–101%), P38 (104–101%), and STAT1 (103–99%) (*P* > 0.05) levels.

As a member of the pro-inflammatory cytokine family, IL-6 may act via the JAK2/STAT3 pathway, and SOCS3 hinders IL-6 signaling with high potency and specificity [27]. As shown in Figure 6C, levels of IL-6 (150%, *P* < 0.001), p-JAK2 (131%, *P* < 0.001), and p-STAT3 (126%, *P* = 0.010) were enhanced with induced α-synuclein expression plus α-synuclein fibrils addition. LM-021 treatment lowered IL-6 level (111%) (*P* = 0.002), and NC009-1 treatment lowered both IL-6 (104%, *P* < 0.001) and p-JAK2 (102%, *P* = 0.001) levels. The reduction of p-JAK2 by LM-021 (117%) and p-STAT3 by both compounds (114–112%) was not statistically significant (*P* > 0.05). There were no changes in total JAK2 (112–105%) and STAT3 (109%) (*P* > 0.05) levels. Furthermore, the reduced SOCS3 upon addition of α-synuclein fibrils (80%, *P* = 0.029) was restored to 103% (LM-021) or 104% (NC009-1) (*P* = 0.044–0.032) with the tested compound treatments. These results showed the anti-inflammatory effects of LM-021 and NC009-1 on PD neuronal cells, and NC009-1 displayed better anti-inflammatory potential.

For GSH biosynthetic route, the first and limiting step is carried out by glutamate-cysteine ligase (GCL), which is composed of catalytic subunit GCLC and modifier subunit GCLM. Glutathione S-transferase pi 1 (GSTP1), a member of large enzyme families, plays an important role in detoxification of reactive electrophilic compounds by conjugation with GSH. As shown in Figure 6D, induced α-synuclein expression plus α-synuclein fibrils addition reduced GCLC (81%, *P* = 0.005) and GSTP1 (75%, *P* = 0.018). Treatment with LM-021 increased the GCLC (102%, *P* = 0.003) and GSTP1 (99%, *P* = 0.026) levels, and treatment with NC009-1 also increased the GCLC (106%, *P* < 0.001) and GSTP1 (98%, *P* = 0.032).

## DISCUSSION

In neurodegenerative diseases, amyloid proteins trigger NLRP3 inflammasome, leading to activation of CASP1 and release of IL-1β [28]. NLRP1 inflammasome also promotes the caspase-1 and IL-1β activation [29]. The expression of CASP1 and IL-1β have been reported in neuronal cells in neurotoxin-induced or transgenic mouse models of PD [30]. α-Synuclein fibrils play a key role in aggravating neuroinflammation. It has been shown that α-synuclein fibrils bind to toll like receptor 2 (TLR2) of primary monocytes to release mature IL-1β via the NLRP3 inflammasome activation [13]. Extracellular α-synuclein activates microglia to produce NO and inflammatory cytokines including IL-1β, IL-6 and TNF-α, which would lead to neuronal death [31]. Furthermore, microglia can be primed by ROS [32], rotenone-induced mitochondrial impairment [33], and 1-methyl-4-phenyl-1,2,3,6-tetrahydropyridine (MPTP) to activate NLRP3 inflammasome [34]. Similarly, in this study, we demonstrated that in the presence of α-synuclein fibrils, BV-2 cells are activated to display increased MHC-II expression, NO release, and IL-1β, IL-6 and TNF-α secretion, and iNOS, NLRP3 and CASP1 expression. The α-synuclein fibrils-mediated inflammatory response is inhibited by two in-house compounds LM-021 and NC009-1 (Figure 3). We further demonstrated that α-synuclein fibrils provoked aggregation, increased oxidative stress, and caused impaired neurite outgrowth and apoptosis via activating NLRP1 inflammasome, IκBα/P65, JNK/JUN and P38/STAT1 pathways downstream to IL-1β binding, and JAK2/STAT3 pathway downstream to IL-6 binding in α-synuclein-expressing BE(2)-M17 cells, and all of these can be ameliorated by LM-021 and NC009-1 (Figures 4–6). Neuroinflammation often interplays with ROS-induced oxidative stress to cause detrimental effects on neurons. Oxidative stress contributing to neurodegeneration in PD has been shown [4]. In this study, we further demonstrated increased ROS production and decreased GSH/GSSG ratio, GCLC and GSPT1 in A53T SNCA-GFP BE(2)-M17 cells, which could be aggravated by addition of α-synuclein fibrils. The oxidative stress with decreased expression of antioxidant enzymes was ameliorated by LM-021 and NC009-1. It is noted that NC009-1 treatment reduced the GSSG levels without increasing the GSH/GSSG ratio. We postulated that the GSSG level was reduced due to decreased oxygen radicals resulted from NC009-1 treatment. However, NC009-1 treatment may not enhance glutathione reductase activity that would catalyse generation of GSH. The cell viability of 85–78% in 100 μM compound-treated BV-2 and BE(2)-M17 cells (Figure 1), indicates the low cytotoxicity of both LM-021 and NC009-1. Taken together, these results suggest potentials of both LM-021 and NC009-1 as agents for treatment of microglia-mediated neurodegeneration in PD. However, *in vitro* models have some limitations to mimic physiological and pathological events in human diseases, such as cell-cell interactions and their behaviours in the brain. It is noted that addition of fibrils versus without did not significantly enhance the pro-inflammatory changes in some assays. However, addition of fibrils did enhance aggregation, increase ASC, IL-1β, p-JNK, p-IκBα, p-P38 and IL-6 expression, and decrease neurite length and branch growth, GSH/GSSG ratio, SOCS3 expression and GSTP1 level in BE(2)-M17 cells expressing A53T α-synuclein. The reason for the inconsistent effects of addition of fibrils on inflammation may be due to the relative short duration of fibrils addition *in vitro*. In our previous study, we did find that the studied compound NC009-1 reduces inflammation in a neurotoxin-induced PD cell model without the addition of fibrils [35], suggesting that the tested compounds may also exert general anti-inflammatory effects. Therefore, *in vivo* experiments including behavioral tests are needed in the future to know if similar results can be recapitulated in animal models.

Previously, we have shown that indole compound NC009-1 displays aggregation-reducing and neuroprotective effects by activating heat shock protein family B (small) member 1 (HSPB1) in tauopathy cell model and spinocerebellar ataxia type 17 cell and mouse models [22, 23]. NC009-1 can also activate apolipoprotein E (APOE) and neurotrophic receptor tyrosine kinase 1 (NTRK1) in Aβ-GFP SH-SY5Y cell and APP_Swe_/PS1_M146V_/Tau_P301L_ triple transgenic mouse models [24]. It is noted that APOE genotype regulates pathology and disease progression in synucleinopathy, with APOE ε4 exacerbating pathology and APOE ε2 protecting against α-synuclein aggregation and neurodegeneration [36]. Overexpression of NTRK1 in peripheral blood mesenchymal stem cells enhances repairing capability in a rat model of PD [37]. Through binding to α-synuclein fibrils, HSPB1 could inhibit fibril growth and cytotoxicity of α-synuclein fibrils [38]. HSPB1 is also essential for the 625 nm photobiomodulation-induced anti-inflammatory function [39]. In addition to the anti-inflammatory and anti-oxidative effects shown in this study, whether NC009-1 could activate HSPB1, APOE and/or NTRK1 expression for neuroprotection in A53T SNCA-GFP BE(2)-M17 cells warrants future studies to clarify further. The underlying mechanisms for anti-inflammatory properties of NC009-1 remain to be determined. However, we propose that it may have COX-inhibiting activity as the other indole derivatives have shown [40, 41]. Since NC009-1 has chemical chaperone activity as shown by thioflavin T assay in the present study and also activate chaperone HSPB1 in our previous study [23], its chaperone activity may reduce α-synuclein aggregation to diminish microglia activation.

Previously, we have shown that coumarin-chalcone derivative LM-021 displays potential in cAMP responsive element binding protein 1 (CREB)-mediated transcriptional activation partially via protein kinase A (PKA) pathway [21]. Elevated level of α-synuclein could inhibit cAMP/PKA-dependent CREB signaling that regulates tyrosine hydroxylase gene expression, leading to dopaminergic neuronal dysfunction [42]. Substantial evidence has shown involvement of PKA/CREB pathway in pathogenesis of PD, and agents augmenting PKA/CREB pathway could protect neurons against MPP^+^-induced decline of mitochondrial membrane potential, oxidative stress and apoptosis in *in vitro*, *ex vivo* and *in vivo* PD models [43, 44]. Upregulation of PKA/CREB in microglia also attenuates activation of microglia and decreases inflammatory damage effects on neurons co-cultured with microglia [45]. Hydrocortisone could stimulate parkin expression via CREB pathway to provide neuroprotective effect in PD cell and mouse models [46]. Under oxidative stress, CREB could be phosphorylated and its transcriptional activity activated to promote neuroprotection [47]. CREB could promote transcription of genes for neuronal survival, neurite outgrowth and neuroplasticity such as brain-derived neurotrophic factor (BDNF) [48], BCL2 apoptosis regulator (BCL2) [49], and growth arrest and DNA damage inducible beta (GADD45B) [50]. Since PKA/CREB suppresses NF-κB signaling and inflammation [45, 51, 52], we proposed that LM-021 may have anti-inflammatory property through activating CREB as our previous study have shown [21]. However, other than the effects shown in this study, future studies will be needed in order to evaluate if LM-021 could enhance CREB and its downstream genes to protect BE(2)-M17 cells against α-synuclein aggregates.

While augmented NLRP3 expression in microglia plays a role in neurodegeneration in PD models [13, 14], different toxic stimuli can activate NLRP1 in neurons [53]. In serum-deprived human CNS neurons, NLRP1 expression is upregulated, leading to proteolytically process of pro-IL-1β into maturation [20]. Studies in murine AD models also show that Aβ oligomers up-regulate NLRP1 inflammasome, which results in cognitive decline associated with the observed neuronal death [54]. In accordance with the previous study, we showed that α-synuclein fibrils activate NLRP1 inflammasome to release mature caspase-1 (Figure 4C) and then promote IL-1β maturation in α-synuclein-expressing BE(2)-M17 neurons (Figure 6A). As inflammasome-mediated caspase-1 activation by certain toxins is known to cause truncation and aggregation of α-synuclein in BE(2)-M17 PD cell model [55], α-synuclein fibrils may initially work like a stimulator or ROS producer that activates NLRP1 inflammasome and IL-1β, which would then further lead to increased α-synuclein aggregates and neurotoxicity in a vicious cycle manner, all of which can be mitigated by LM-021 and NC009-1 in the present study (Figures 4–6). However, further *in vivo* studies are necessary to strengthen the potential of LM-021 and NC009-1 as agents for treatment of α-synuclein-mediated neurodegeneration in PD.

Deviation from the strict control of MAPK signaling pathway has been implicated in PD [56]. Increased IL-1β in the brains of human PD and mouse PD models have been reported [57, 58]. Reacting to IL-1β binding signaling, the downstream JNK or P38 is phosphorylated and translocated to the nucleus to activate transcription factor JUN or STAT1, which subsequently promotes the expression of pro-apoptotic genes [59, 60]. Proinflammatory cytokines and p-P38 are up-regulated by 6-OHDA and agents suppressing p-P38 provide neuroprotection in a PD rat model [61]. In MPTP-, lipopolysaccharide- and rotenone-induced PD animal models, polyphenols exert anti-inflammatory effects through inhibiting p-P65 nuclear translocation and activation [62]. In the present study, the IL-1β-induced downstream pathways including p-IκBα/p-P65, p-JNK/p-JUN and p-P38/p-STAT1 in the BE(2)-M17 cells were augmented by α-synuclein fibrils and attenuated by LM-021 or NC009-1 (Figure 6B), indicating the anti-inflammatory effects of LM-021 and NC009-1 via targeting IL-1β and its downstream signaling in A53T SNCA-GFP BE(2)-M17 cells. However, the direct evidence that both compounds are targeting IL-1β as potential mechanism of action is not shown, which is one of the limitations of this study. Therefore, further studies using cellular models treated with IL-1β to evaluate if this selective IL-1β activation is inhibited by the tested compounds are necessary in the future.

Continuous deregulated synthesis of IL-6 plays a critical role in chronic inflammation [63]. It is well known that in acute phase, IL-6 plays a key role in activating microglia within the brain. The pro-inflammatory effect of IL-6 can be mediated through activation of STAT3 by JAK2, whereas SOCS3 acts against JAK2/STAT3 signaling to counteract the IL-6-mediated inflammation [27]. Increased IL-6 in the brains, CSF and plasma of human PD, and brains of PD mouse models suggest that IL-6 may be involved in inflammation in PD [57, 64, 65]. In a PD mouse model induced by α-synuclein overexpression, miR-let-7a overexpression via injection of miR-7 mimics into mouse striatum can suppress the enhanced STAT3 to reduce neuroinflammation [66]. Similarly, our study results show that α-synuclein fibrils increased IL-6 and JAK2/STAT3 and decreased SOCS3, both of which can be ameliorated by LM-021 and NC009-1 (Figure 6C), indicating the anti-inflammatory effects of LM-021 and NC009-1 via targeting IL-6/JAK2/STAT3 signaling in A53T SNCA-GFP BE(2)-M17 cells.

## CONCLUSIONS

In summary, our study shows that in addition to microglia activation and pro-inflammatory cytokine release, α-synuclein fibrils induce neuronal inflammation that contributes to increased aggregation, oxidative stress, and neurotoxicity in A53T SNCA-GFP BE(2)-M17 cells. LM-021 and NC009-1 reduce aggregation, neuroinflammation, ROS and apoptosis, elevate anti-oxidative enzymes, and promote neurite outgrowth by downregulating NLRP1-, IL-1β- and IL-6-mediated pathways and their downstream caspase-1, IκBα/P65, JNK/JUN, P38/STAT1 and JAK2/STAT3 signaling. The study results provide insight into the involvement of neuroinflammation in PD pathogenesis and the potential of LM-021 and NC009-1 in treating PD.

## MATERIALS AND METHODS

### Compounds

Coumarin-chalcone hybrid (E)-3-(3-(4-(dimethylamino)phenyl)acryloyl)-4-hydroxy-2H-chromen-2-one (LM-021) and indole derivative (E)-3-((1H-indol-3-yl)methyl)-4-(2-nitrophenyl)but-3-en-2-one (NC009-1) were synthesized and analyzed as described [67–69]. Kaempferol and trehalose were purchased from Sigma-Aldrich (St. Louis, MO, USA).

### Bioavailability and BBB permeation prediction

MW, HBD, HBA, cLopP, and PSA of LM-021 and NC009-1 were analyzed and calculated using ChemDraw (http://www.perkinelmer.com/tw/category/chemdraw). Oral bioavailability and BBB penetration were predicted based on Lipinski’s criteria (MW ≤450, HBD ≤5, HBA ≤10, cLogP ≤5) [70] and PSA (<90 Å^2^) [71]. In addition, prediction of BBB permeability was carried out by using online BBB predictor (https://www.cbligand.org/BBB/) (threshold of 0.02) [72].

### Free radical-scavenging and oxygen radical antioxidant capacity (ORAC) assays

The potential radical-scavenging activity of test compounds was assayed using stable free radical DPPH (Sigma-Aldrich). Briefly, kaempferol (a positive control) [73], LM-021 or NC009-1 (10–160 μM) was added to DPPH (100 μM in 99% ethanol). After mixing with vortex for 15 s and standing at room temperature for 30 min, the scavenging capacity was quantified at optical density (OD) 517 nm using a Multiskan GO microplate spectrophotometer (Thermo Fisher Scientific, Waltham, MA, USA). Radical-scavenging activity was calculated by using the equation: 1 – (absorbance of sample/absorbance of control) × 100%, and half maximal effective concentration (EC50) estimated using the linear interpolation method.

The ORAC assay was performed using OxiSelect™ kit according to the manufacturer’s instruction (Cell Biolabs, San Diego, CA, USA). Briefly, fresh dilutions of trolox standards (2.5 to 50 μM) in 50% acetone were prepared. Test compounds were also diluted with 50% acetone. Blank (50% acetone), standards and samples were mixed with fluorescein and incubated at 37° C for 30 min. Following given free radical initiator 2,2’-azobis(2-methylpropionamidine) dihydrochloride (AAPH), the produced peroxyl radicals (ROO•) quench the fluorescent probe over time [74]. The course of the reaction was recorded for 60 min, with one measurement every 5 min (FLx800, Bio-Tek). The excitation and emission wavelengths were set at 480 nm and 520 nm, respectively. To quantify the oxygen radical antioxidant activity in a sample, the area under the curve (AUC) for blank, standard and samples were calculated. After subtraction of the blank, the equivalent trolox concentrations of samples were expressed based on the trolox standard curve.

### Cell culture and compound cytotoxicity assay

Mouse BV-2 microglial cells were kept in Dulbecco’s modified Eagle medium (DMEM) added with 10% fetal bovine serum (FBS) (Invitrogen, Carlsbad, CA, USA). The BV-2 microglial cell was generously given by Dr. Han-Min Chen, Catholic Fu-Jen University, New Taipei City, Taiwan. Human neuroblastoma BE(2)-M17 (from American Type Culture Collection, CRL-2267) were maintained in DMEM/Nutrient mixture F-12 (DMEM/F-12) added with 10% FBS. Cells were cultured in an incubator at 37° C (NuAire, Plymouth, MN, USA), 95% relative humidity and 5% CO_2_.

To evaluate compound cytotoxicity, BV-2 (2 × 10^4^) or BE(2)-M17 (6 × 10^4^) cells were plated in 48-well dishes, grown for 20 h, and treated with the test compounds (0.1−100 μM). Next day, the cells were treated with 20 μl of tetrazolium dye MTT (3-(4,5-dimethylthiazol-2-yl)-2,5-diphenyltetrazolium bromide, 5 mg/ml) (Sigma-Aldrich) at 37° C for 4 h, followed by addition of 200 μl lysis buffer (10% Triton X-100, 0.1 N HCl, 18% isopropanol) for 16 h to dissolve the insoluble purple formazan. The absorbance of the colored solution was measured at OD 570 nm by using the FLx800 fluorescence microplate spectrophotometer (Bio-Tek, Winooski, VT, USA).

### Biochemical examination and quantification of α-synuclein aggregates

SNCA-His proteins were prepared as previously described [75]. The purified SNCA-His protein (the final concentration, 20 μM) was continuously shaken in phosphate-buffered saline (PBS) at 37° C for 3 days. For examination of α-synuclein aggregates, the formed fibrils were placed on a 200-Mesh copper (holey-carbon) grid and immerged into liquid nitrogen. For cryo-TEM examination, samples were inserted into a CP3 cryoholder (Gatan, Inc., Pleasanton, CA, USA) and observed at 200 kV by JEM-2100F transmission electron microscope (JEOL, Tokyo, Japan). Images were captured by using a 1024 × 1024 CCD camera (Gatan). For α-synuclein aggregate quantification, thioflavin T (10 μM final concentration; Sigma-Aldrich), a dye exhibiting enhanced fluorescence upon binding to amyloid fibrils [76], was added to the aggregated α-synuclein (± trehalose, LM-021 or NC009-1; 1−10 μM) and incubated for 5 min at room temperature. Trehalose was included as a positive control for inhibiting α-synuclein amyloid fibril formation [75]. Dimethyl sulfoxide in 1% was used as a negative control. Thioflavin T fluorescence intensity of samples was recorded by using a microplate reader (Bio-Tek FLx800), with a combination of excitation at 420/50 nm and emission at 485/20 filter.

### Activation of BV-2 microglia and detection of inflammatory mediators

BV-2 cells (3 × 10^5^) were plated into 6-well dishes, grown for 28 h and stimulated with α-synuclein fibrils (2 μM) for 20 h. To examine anti-inflammatory potential of the test compounds, BV-2 cells were pretreated with LM-021 or NC009-1 (10 μM) for 8 h before α-synuclein fibrils stimulation. On day 3, the cells were fixed with 4% paraformaldehyde, permeabilized with 0.1% Triton X-100, blocked with 2% bovine serum albumin (BSA), and stained with primary anti-MHC-II antibody (1:1000; Invitrogen) at 4° C overnight, followed by subjecting to secondary Cy5-goat anti-mouse (IgG) antibody (1:1000; Invitrogen) for 2 h at room temperature. Nuclei were stained with 4’,6-diamidino-2-phenylindole (DAPI; 0.1 μg/ml; Sigma-Aldrich). The stained cells were imaged by using a Zeiss LSM 880 confocal laser scanning microscope (Zeiss, Oberkochen, Germany). In addition, the release of NO in cell culture medium was quantified by using the Griess reagent kit (Thermo Fisher Scientific). The levels of IL-1β, IL-6 and TNF-α in medium were determined by multiplex assay (Inflammation Core Facility of Institute of Biomedical Sciences, Academia Sinica, Taipei, Taiwan). Levels of NLRP3, CASP1, iNOS, IL-1β, IL-6 and TNF-α proteins in BV-2 cells were examined by Western blot using α-tubulin as a loading control as described below.

### Wild type and A53T SNCA-GFP BE(2)-M17 cells

To generate human cell line with inducible α-synuclein expression, BE(2)-M17 dopaminergic neuroblastoma cells were transduced by the lentivirus bearing wild type or A53T SNCA (NM_000345)-GFP (AAB02574) [75]. BE(2)-M17 cells were overlaid onto 6-well (5 × 10^5^/well) dishes, grown for 20 h, and transduced by lentivirus (multiplicity of infection: 0.01) (National RNAi Core Facility, Academia Sinica, Taiwan). Polybrene (hexadimethrine bromide, 8 μg/ml final concentration; Sigma-Aldrich) was included during transduction to increase the efficiency of retrovirus-mediated gene transfer. Six hours later, the medium was replaced with fresh medium. Next day, the infected cells were passaged onto a 10-cm dish added with blasticidin (10 μg/ml; InvivoGen, San Diego, CA, USA). The blasticidin-containing medium was replaced every 2 to 3 days until a pool of blasticidin-resistant cells was generated (about 3–4 weeks). The clones were selected, expanded, and examined for doxycycline (5 μg/ml; Sigma-Aldrich)-induced SNCA-GFP expression using anti-α-synuclein and anti-GFP antibodies as described below.

### Caspase-1 and LDH assays

As described, SNCA-GFP BE(2)-M17 cells were seeded in 6-well (2 × 10^5^/well), added with retinoic on day 1, and treated with tested compounds, doxycycline and α-synuclein fibrils on day 2. On day 7, cells were collected and lysed by six freeze/thaw cycles. After centrifugation, caspase-1 activity in 50 μg cell extracts was quantified using caspase-1 fluorimetric assay kit (BioVision, Milpitas, CA, USA). The mixture was incubated for 2 h at 37° C, and caspase-1 activity was determined at 420/50 nm excitation and 485/20 nm emission wavelengths (FLx800 fluorescence microplate reader, Bio-Tek). In addition, the LDH released into culture medium was measured by using LDH cytotoxicity assay kit (Cayman, Ann Arbor, MI, USA). The absorbance was read at 490 nm with Multiskan GO microplate reader.

### Cellular reactive oxygen species analysis

For ROS analysis, SNCA-GFP BE(2)-M17 cells were plated onto 96-well (1 × 10^4^/well) and treated with retinoic acid, tested compounds, doxycycline and α-synuclein fibrils as described. On day 7, cell-permeant CellROX Deep Red reagent (5 μM; Molecular Probes, Eugene, OR, USA) and Hoechst 33342 (0.1 μg/ml; Sigma-Aldrich) were administered to the cells and incubated at 37° C for 30 min. ROS in cells was analyzed using the high content analysis system, at excitation 631/28 nm and emission 692/40 nm wavelengths (ImageXpress Micro Confocal; Molecular Devices).

### Total and reduced GSH measurement

Cells were re-dispensed in ice-cold lysis buffer (0.5% nonyl phenoxypolyethoxylethanol in PBS). Samples were deproteinized by trichloroacetic acid and neutralized by sodium hydrogen carbonate. GSH/GSSG ratio was measured using the GSH/GSSG ratio detection assay kit II (Abcam, Cambridge, UK) following the manufacturer’s instructions. Upon reacting with GSH, the non-fluorescent green dye used in this assay becomes fluorescent. Total and reduced GSH levels were measured with 485/20 nm excitation and 528/20 nm emission wavelengths (FLx800, Bio-Tek). Absolute GSH and GSSG levels were quantified using standard curves of GSH and GSSG.

### α-Synuclein aggregation analysis

SNCA-GFP BE(2)-M17 cells were seeded in the presence of retinoic acid (5 μM) in 12-well (5 × 10^4^/well) plate on day 1. On day 2, cells were added with LM-021 or NC009-1 (10 μM) for 8 h, and then SNCA-GFP expression induced with doxycycline (5 μg/ml). In addition, the cells were treated with 0.1 μM preformed α-synuclein fibrils to seed the aggregate formation of induced SNCA-GFP [77]. Culture medium was replaced with fresh medium containing retinoic acid, doxycycline, α-synuclein fibrils and compound every 2–3 days for 5 days. Then cells were fixed, permeated, and stained with ProteoStat® dye (1/5000; Enzo Life Sciences, Farmingdale, NY, USA). After nuclei staining with DAPI (1:5000), cell images were captured and percentages of aggregated cells were calculated by evaluating overlapped green (GFP, 482/35-nm excitation and 536/40-nm emission) and red (ProteoStat®, 543/22-nm excitation and 593/40-nm emission) fluorescence signals (ImageXpress Micro Confocal High-Content Imaging System; Molecular Devices, San Jose, CA, USA).

Filter trap assay was used to quantify α-synuclein aggregates in cell lysates. Protein (5 μg) was diluted in 2% SDS in PBS and filtered through a cellulose acetate membrane (0.2-μm pore size; Merck, Kenilworth, NJ, USA) pre-equilibrated in 2% SDS in PBS on a slot-blot equipment (GE Healthcare, Chicago, IL, USA). The membrane was washed 3 times with 2% SDS buffer, blocked in PBS containing 5% BSA and then stained with anti-GFP antibody (1:500; Santa Cruz Biotechnology, Santa Cruz, CA, USA). The immune complexes on the filter were detected using goat anti-mouse IgG-horseradish peroxidase (HRP) antibody (1:5000; GeneTex, Irvine, CA, USA) and chemiluminescent substrate (Millipore, Temecula, CA, USA).

### Neurite outgrowth analysis

For neurite outgrowth analysis, SNCA-GFP BE(2)-M17 cells were seeded in 24-well plate (2 × 10^4^/well) in the presence of retinoic acid on day 1, and treated with tested compounds, doxycycline and α-synuclein fibrils on day 2 as described. On day 7, cells were fixed, permeated, and finally stained with neuronal class III β-tubulin (TUBB3) antibody (1:1000; Covance, Princeton, NJ, USA), followed by anti-rabbit Alexa Fluor® 555 antibody (1:1000; Thermo Fisher Scientific). After nuclei staining, neuronal images were captured at excitation 531/40 nm and emission 593/40 nm wavelengths using an ImageXpress Micro Confocal High-Content Imaging System (Molecular Devices). Neurite total length (μm) and numbers of process (primary neurites originated from neuronal cell body) and branch (secondary neurites extended from primary neurites) were analyzed (Neurite Outgrowth Application Module; MetaXpress High-Content Image Acquisition and Analysis Software, Molecular Devices). For each sample, around 3000 cells were analyzed in each of three independent experiments.

### Western blot analysis

Cells were lysed in buffer (50 mM Tris-HCl pH 8.0, 150 mM NaCl, 1 mM EDTA pH 8.0, 0.1% SDS, 0.5% sodium deoxycholate, 1% Triton X-100) comprising the protease and phosphatase inhibitor cocktail (Sigma-Aldrich). The lysates were sonicated and centrifuged at 12,000 × g for 10 min at 4° C and protein concentrations determined (protein assay kit; Bio-Rad, Hercules, CA, USA). Total proteins (20 μg) were electrophoresed on 10% SDS-polyacrylamide gel and transferred onto polyvinylidene difluoride membrane (Sigma-Aldrich) by reverse electrophoresis. After blocking, the membrane was subjected to staining with each of the following primary antibodies, α-synuclein (1:1000; BD Biosciences, Franklin Lakes, NJ, USA), GFP (1:500; Santa Cruz Biotechnology), NLRP1 (1:1000; Novus Biologicals, Centennial, CO, USA), NLRP3 (1:1000; Cell Signaling, Danvers, MA, USA), ASC (1:1000; Abcam), CASP1 (1:1000; Cell Signaling), iNOS (1:1000; Cell Signaling), IL-1β (1:1000; Abcam), IL-6 (1:1000; Abcam), TNF-α (1:1000; Abcam), IκBα (NF-κB inhibitor α) (1:1000; Cell Signaling), p-IκBα (S32/36) (1:1000; Cell Signaling), P65 (RELA proto-oncogene, NF-κB subunit) (1:1000; Cell Signaling), p-P65 (S536) (1:1000; Cell Signaling), JNK (MAPK8) (1:1000; Cell Signaling), p-JNK (T183/Y185) (1:500; Cell Signaling), JUN (Jun proto-oncogene, AP-1 transcription factor subunit) (1:1000; Cell Signaling), p-JUN (S63) (1:1000; Cell Signaling), P38 (MAPK14) (1:1000; Cell Signaling), p-P38 (T180/Y182) (1:1000; Cell Signaling), STAT1 (signal transducer and activator of transcription 1) (1:1000; Cell Signaling), p-STAT1 (S727) (1:1000; Cell Signaling), JAK2 (Janus kinase 2) (1:1000; Cell Signaling), p-JAK2 (Y1007/1008) (1:1000; Invitrogen), STAT3 (signal transducer and activator of transcription 3) (1:500; Santa Cruz Biotechnology), p-STAT3 (Y705) (1:500; Santa Cruz Biotechnology), SOCS3 (suppressor of cytokine signaling 3) (1:500; Santa Cruz Biotechnology), GCLC (glutamate-cysteine ligase catalytic subunit) (1:1000; Abcam), GSTP1 (glutathione S-transferase pi 1) (1:1000; Abcam), α-tubulin (1:1000; Sigma-Aldrich), or glyceralde-hyde-3-phosphate dehydrogenase (GAPDH) (1:5000; MDBio, Taipei, Taiwan) at room temperature 2 h or 4° C overnight. The immune complexes were detected using secondary antibody, goat anti-mouse or goat anti-rabbit IgG-HRP (1:5000; GeneTex) and chemiluminescent substrate (Millipore). Immunoreactive bands were captured using the ImageQuant™ LAS 4000 (GE Healthcare, Chicago, IL, USA). The resulting bands were quantified using Multi Gauge V3.0 software (Fijifilm, Tokyo, Japan). Levels of the indicated proteins were normalized with an internal control (GAPDH or β-Tubulin).

### Statistical analysis

Three independent experiments were conducted to get a data set and data were expressed as the means ± standard deviation (SD). Groups were compared by using one-way analysis of variance (ANOVA) with a post hoc Tukey test where appropriate (comparing several groups). All *P* values were two-tailed, and values lower than 0.05 considered being statistically significant.

## Supplementary Material

Supplementary Materials

Supplementary Figures
