# Supplementary material for: Coumarin-chalcone hybrid LM-021 and indole derivative NC009-1 targeting inflammation and oxidative stress to protect BE(2)-M17 cells against α-synuclein toxicity
Source: Aging (Albany NY). 2023 Aug 11;15(16):8061–89. doi: 10.18632/aging.204954 (PMC10497001; doi:10.18632/aging.204954)
Supplement: Supplementary Materials [file aging-15-204954-s001.pdf]

SUPPLEMENTARY MATERIALS

Supplementary Materials

BE(2)-M17 cells with induced wild type or A53T SNCA-GFP expression

Lentivirus containing in-frame wild type (WT) or A53T [1] green fluorescent protein-tagged α-synuclein (SNCA-GFP) (Supplementary Figure 1A) was used to transduce human neuroblastoma BE(2)-M17 cells [2]. The selected blasticidin-resistance clones WT-4, WT-5, A53T-8 and A53T-11 were expanded, differentiated with retinoic acid [3], and examined for induced SNCA-GFP expression after doxycycline treatment for 5 days (Supplementary Figure 1B). The expressed SNCA-GFP fusion proteins (50 kDa) were confirmed by GFP images (Supplementary Figure 1C) and immunoblotting using GFP and α-synuclein antibodies (Supplementary Figure 1D). Compared to the endogenous hypoxanthine phosphoribosyltransferase 1 (HPRT1), 25–62 folds SNCA-GFP mRNA were induced by doxycycline in these clones (Supplementary Figure 1E).

α-Synuclein aggregation and neurite outgrowth reduction in α-synuclein fibrils-treated wild type/ A53T SNCA-GFP BE(2)-M17 cells

α-Synuclein aggregation and neurite outgrowth of retinoic acid-differentiated wild type and A53T SNCA-GFP BE(2)-M17 cells were examined after inducing α-synuclein expression in the presence or absence of preformed α-synuclein fibrils for five days (Supplementary Figure 2A). ProteoStat® [4] was used to measure α-synuclein aggregation by HCA. Addition of preformed α-synuclein fibrils significantly provoked aggregation of both wild type and A53T SNCA-GFP in BE(2)-M17 cells (from 7.1–8.3% to 19.5–23.6%; $p < 0.001$) (Supplementary Figure 2B). When protein samples from these cells were subjected to filter trap assay using GFP antibody, both wild type and A53T α-synuclein-containing insoluble aggregates were greatly increased (from 7.4–8.5% to 100.0–103.7%; $p < 0.001$) upon addition of preformed α-synuclein fibrils (Supplementary Figure 2C).

α-Synuclein plays a vital role in regulating neurite outgrowth [5, 6]. We examined the neurite total length, process (primary neurite, a projection from the cell body of a neuron) and branch (an extension from primary neurite) of these cells. As shown in Supplementary Figure 2D, addition of preformed α-synuclein fibrils to BE(2)-M17 cells led to significant reduction of neurite total length (from 24.3 μm to 19.3 μm; $p < 0.001$). In addition, induction of wild type or A53T α-synuclein expression significantly reduced neurite total length (from 24.3 μm to 17.1–13.0 μm; $p < 0.001$) and branch (from 1.05 to 0.85–0.60; $p = 0.036$–<0.001) compared to BE(2)-M17 cells. Significantly reduced neurite total length (from 19.3 μm to 15.8–11.5 μm, $p < 0.001$) and branch (from 0.94 to 0.68–0.49; $p = 0.003$–<0.001) were also observed in α-synuclein-expressing cells with preformed α-synuclein fibrils addition compared to BE(2)-M17 cells with preformed α-synuclein fibrils treatment. Neuronal process was significantly reduced by induction of A53T α-synuclein in BE(2)-M17 cells (from 1.85 to 1.58–1.54; $p = 0.009$–0.002) or addition of preformed α-synuclein fibrils to A53T α-synuclein-expressing BE(2)-M17 cells (from 1.79 to 1.56–1.49; $p = 0.029$–0.003).

Neuroprotective effects LM-021 and NC009-1 in A53T-11 SNCA-GFP BE(2)-M17 cells

To evaluate neuroprotective effects LM-021 and NC009-1 in A53T-11 SNCA-GFP BE(2)-M17 cells, LDH, caspase-1 and ROS were examined (Supplementary Figure 3A). As shown in Supplementary Figure 3B, 3C, A53T α-synuclein overexpression plus preformed α-synuclein fibrils addition increased LDH release (120%, $P = 0.005$) and caspase-1 (117%, $P = 0.044$) activities, and LM-021 and NC009-1 treatments reduced LDH release (from 120% to 106–87%; $P = 0.045$–<0.001) and caspase-1 (from 117% to 91–81%; $P = 0.003$–<0.001) in A53T-11 cells. In addition, A53T α-synuclein overexpression plus preformed α-synuclein fibrils addition increased cellular ROS (126%; $P = 0.002$), and both LM-021 and NC009-1 treatments significantly reduced ROS (from 126% to 105–104%; $P = 0.006$–0.005) (Supplementary Figure 3D).

The effects LM-021 and NC009-1 on α-synuclein aggregation were assessed by filter trap assay and ProteoStat® stain. Application of LM-021 and NC009-1 led to significant aggregation reduction (from 100% to 64–62%; $P = 0.008$–0.006) (Supplementary Figure 4A). Quantification of percentage of aggregated cells also revealed 6–7% reduction of aggregated cells (from 17% to 13–12%; $P = 0.035$–0.022) (Supplementary Figure 4B). In consistent with the effects on α-synuclein aggregation, LM-021 and NC009-1 treatments significantly promoted neurite outgrowth in A53T-11 SNCA-GFP BE(2)-M17 cells (length: from 9.7 μm to 13.8–14.5 μm, process: from 1.45 to 1.69–1.75, branch: from 0.32 to 0.65–0.69; $P < 0.001$) (Supplementary Figure 4C).

MATERIALS AND METHODS

Real-time PCR assay

To measure the induced SNCA-GFP expression in wild type and A53T SNCA-GFP BE(2)-M17 cells, total RNA was purified using TRIzol reagent (Invitrogen), treated with DNase I (Stratagene, La Jolla, CA, USA) to eliminate genomic DNA. Subsequently cDNA was synthesized using reverse transcriptase (Thermo Fisher Scientific). Relative SNCA-GFP mRNA was determined in 50 ng cDNA by real-time PCR (StepOnePlus™ Real-time PCR system; Applied Biosystems, Foster City, CA, USA) with customized GFP primers (forward primer: 5'-GAGCGCACCATCTTCTTCAAG-3', reverse primer: 5'-TGTCGCCCTCGAACTTCAC-3') and FAM/NFQ probe (5'-ACGACGGCAACTACA-3'), and TaqMan HPRT1 (NM_000194) endogenous control (VIC/MGB probe, 4326321E) (Applied Biosystems). Fold change was determined using the formula 2ΔCt, ΔCT = CT (HPRT1) – CT (GFP), in which CT indicates cycle threshold.

REFERENCES

1. Polymeropoulos MH, Lavedan C, Leroy E, Ide SE, Dehejia A, Dutra A, Pike B, Root H, Rubenstein J, Boyer R, Stenroos ES, Chandrasekharappa S, Athanassiadou A, et al. Mutation in the alpha-synuclein gene identified in families with Parkinson's disease. Science. 1997; 276:2045–7. https://doi.org/10.1126/science.276.5321.2045 PMID:9197268

2. Andres D, Keyser BM, Petrali J, Benton B, Hubbard KS, McNutt PM, Ray R. Morphological and functional differentiation in BE(2)-M17 human neuroblastoma cells by treatment with Trans-retinoic acid. BMC Neurosci. 2013; 14:49. https://doi.org/10.1186/1471-2202-14-49 PMID:23597229

3. Filograna R, Civiero L, Ferrari V, Codolo G, Greggio E, Bubacco L, Beltramini M, Bisaglia M. Analysis of the Catecholaminergic Phenotype in Human SH-SY5Y and BE(2)-M17 Neuroblastoma Cell Lines upon Differentiation. PLoS One. 2015; 10:e0136769. https://doi.org/10.1371/journal.pone.0136769 PMID:26317353

4. Oshinbolu S, Shah R, Finka G, Molloy M, Uden M, Bracewell DG. Evaluation of fluorescent dyes to measure protein aggregation within mammalian cell culture supernatants. J Chem Technol Biotechnol. 2018; 93:909–17. https://doi.org/10.1002/jctb.5519 PMID:29540956

5. Lee HJ, Lee K, Im H. α-Synuclein modulates neurite outgrowth by interacting with SPTBN1. Biochem Biophys Res Commun. 2012; 424:497–502. https://doi.org/10.1016/j.bbrc.2012.06.143 PMID:22771809

6. Kim S, Lim J, Bang Y, Moon J, Kwon MS, Hong JT, Jeon J, Seo H, Choi HJ. Alpha-Synuclein Suppresses Retinoic Acid-Induced Neuronal Differentiation by Targeting the Glycogen Synthase Kinase-3β/β-Catenin Signaling Pathway. Mol Neurobiol. 2018; 55:1607–19. https://doi.org/10.1007/s12035-016-0370-9 PMID:28190238
