# Supplementary material for: Coumarin-chalcone hybrid LM-021 and indole derivative NC009-1 targeting inflammation and oxidative stress to protect BE(2)-M17 cells against α-synuclein toxicity
Source: Aging (Albany NY). 2023 Aug 11;15(16):8061–89. doi: 10.18632/aging.204954 (PMC10497001; doi:10.18632/aging.204954)
Supplement: Supplementary Figures [file aging-15-204954-s002.pdf]

SUPPLEMENTARY FIGURES

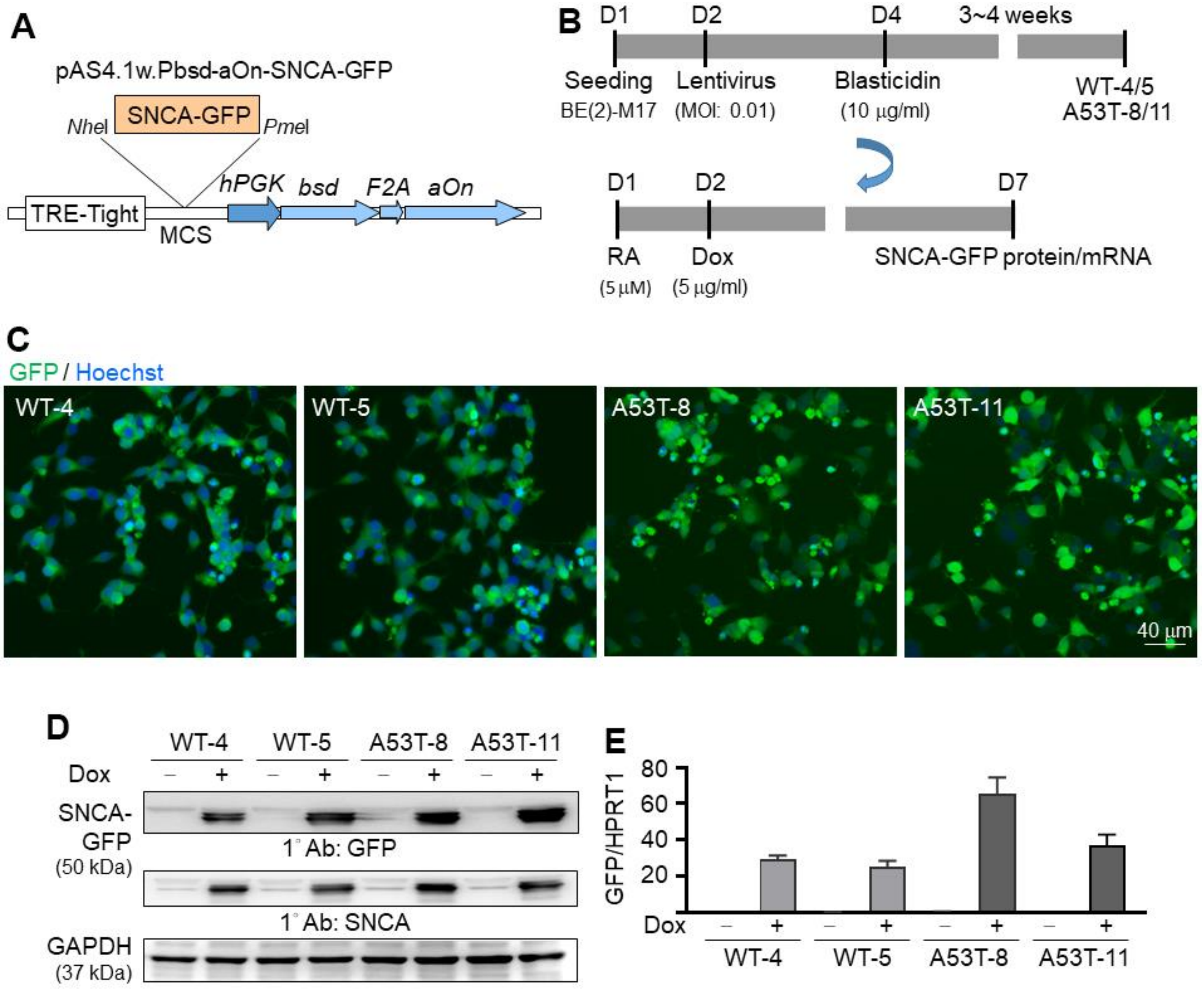

Supplementary Figure 1. Wild type and A53T SNCA-GFP BE(2)-M17 cells. (**A**) Lentiviral vector with wild type (WT) or A53T SNCA-GFP cloned between *Nhe*I and *Pme*I sites in multiple cloning site (MCS) and driven by TRE-Tight promoter. The in-frame fused *bsd* (blasticidin, selective marker)-*aOn* (transcription factor, activating TRE-Tight) is under the control of human phosphoglycerate kinase (*hPGK*) promoter. The fusion protein is cleaved by *F2A* protease into functional bsd and aOn. (**B**) Experimental flow chart. BE(2)-M17 cells were seeded on day 1, transduced at 0.01 MOI with lentivirus carrying WT or A53T SNCA-GFP on day 2, and selected with blasticidin (10 µg/ml) on day 4 for 3–4 weeks. The resultant blasticidin-resistant WT-4/5 and A53T-8/11 clones were expanded. Following neuronal differentiation of the expanded blasticidin-resistant cells with retinoic acid (RA, 5 µM) on day 1, SNCA-GFP expression was induced with doxycycline (Dox, 5 µg/ml) on day 2, and SNCA-GFP image, protein and mRNA examined on day 7. (**C**) GFP images of WT-4/5 and A53T-8/11 cells. Nuclei were detected with Hoechst 33342 (blue). (**D**) Western blot analysis of SNCA-GFP protein probed with GFP and α-synuclein (SNCA) antibodies, using GAPDH as a loading control. (**E**) SNCA-GFP mRNA relatively to HPRT1 mRNA was determined by quantitative real-time PCR.

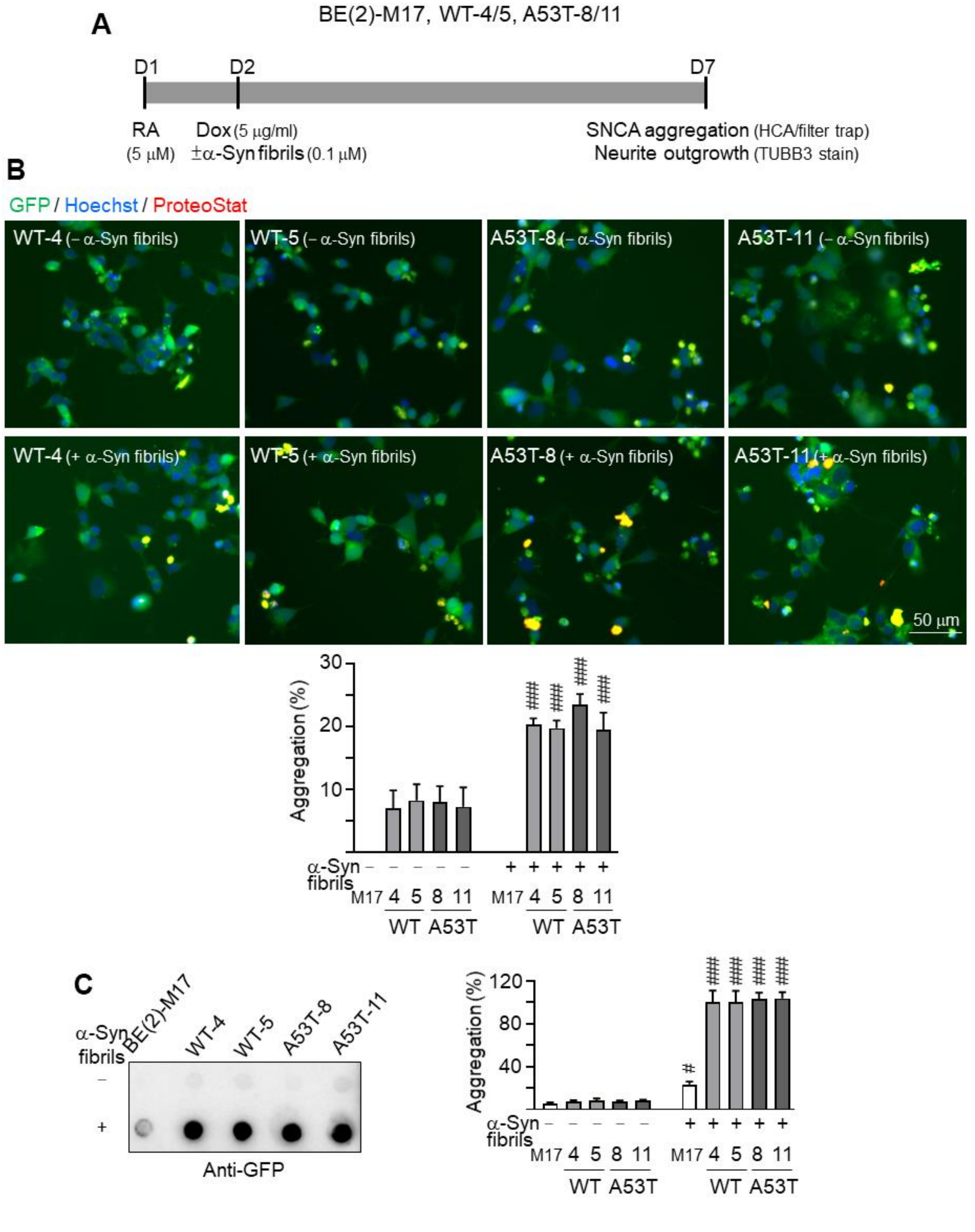
A
BE(2)-M17, WT-4/5, A53T-8/11
D1
D2
D7
RA (5 μM)
Dox (5 μg/ml) ±α-Syn fibrils (0.1 μM)
SNCA aggregation (HCA/filter trap)
Neurite outgrowth (TUBB3 stain)
B
GFP / Hoechst / ProteoStat
WT-4 (– α-Syn fibrils)
WT-5 (– α-Syn fibrils)
A53T-8 (– α-Syn fibrils)
A53T-11 (– α-Syn fibrils)
WT-4 (+ α-Syn fibrils)
WT-5 (+ α-Syn fibrils)
A53T-8 (+ α-Syn fibrils)
A53T-11 (+ α-Syn fibrils)
50 μm
Aggregation (%)
α-Syn fibrils
M17 4 5 8 11
WT A53T
C
α-Syn fibrils
BE(2)-M17
WT-4
WT-5
A53T-8
A53T-11
Anti-GFP
Aggregation (%)

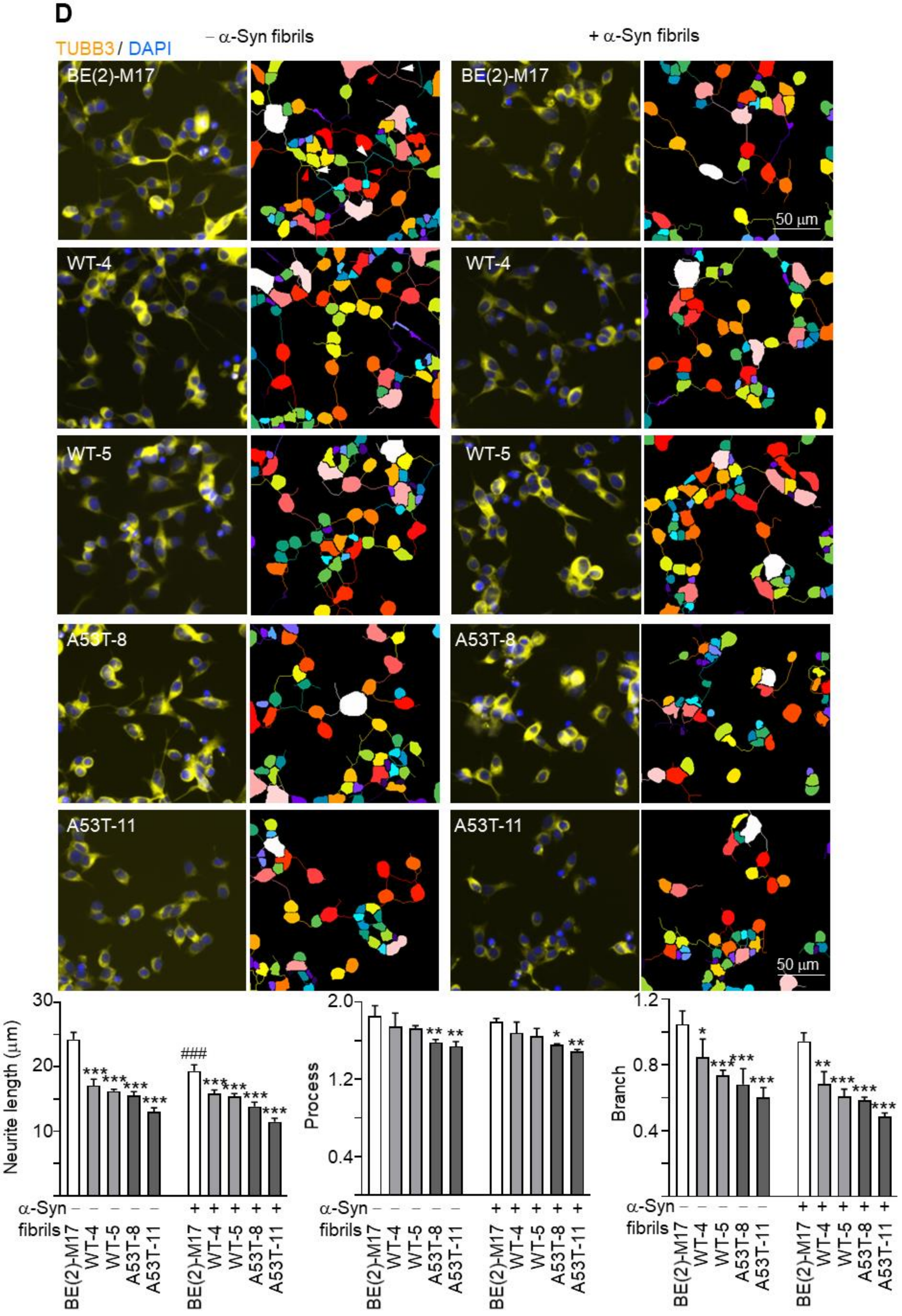

Supplementary Figure 2. α-Synuclein aggregation and neurite outgrowth analyses on wild type and A53T SNCA-GFP BE(2)-M17 cells. (**A**) Experimental flow chart. Cells were seeded with retinoic acid (RA, 5 μM) on day 1. On day 2, SNCA-GFP expression was

induced with doxycycline (Dox, 5 μg/ml), followed by addition of preformed α-synuclein fibril (0.1 μM). On day 7, high content analysis (HCA) and filter trap assay of α-synuclein aggregation as well as neurite outgrowth analysis were performed. (**B**) Fluorescent microscopy images of WT-4/5 and A53T-8/11 SNCA-GFP-expressing cells (green) without (top panel) or with (bottom panel) preformed fibril addition. Nuclei were detected with Hoechst 33342 (blue) and overlapped SNCA-GFP and ProteoStat®-stained (red) aggregates marked (yellow). Quantification of percentage of aggregated cells was shown below (n = 3). (**C**) α-Synuclein aggregates measured by filter trap assay using a GFP antibody. To normalize, the relative α-synuclein aggregate with fibril addition in WT-4 cells was set as 100%. (**D**) Fluorescent microscopy images of BE(2)-M17 and WT-4/5, A53T-8/11 SNCA-GFP cells stained with TUBB3 for neurite outgrowth quantification without (left panel) or with (right panel) preformed fibril addition, with nuclei detected (DAPI, blue). Also shown were images of the neurites and cell bodies being outlined for neurite outgrowth quantification. In uninduced cells, processes and branches are indicated with red and white arrows, respectively. Quantification of neurite length, brunch and process was shown below (n = 3). *P* values: comparisons between with and without fibrils addition (#: $P < 0.05$, ###: $P < 0.001$), or between BE(2)-M17 and SNCA-GFP cells (*: $P < 0.05$, **: $P < 0.01$, ***: $P < 0.001$).

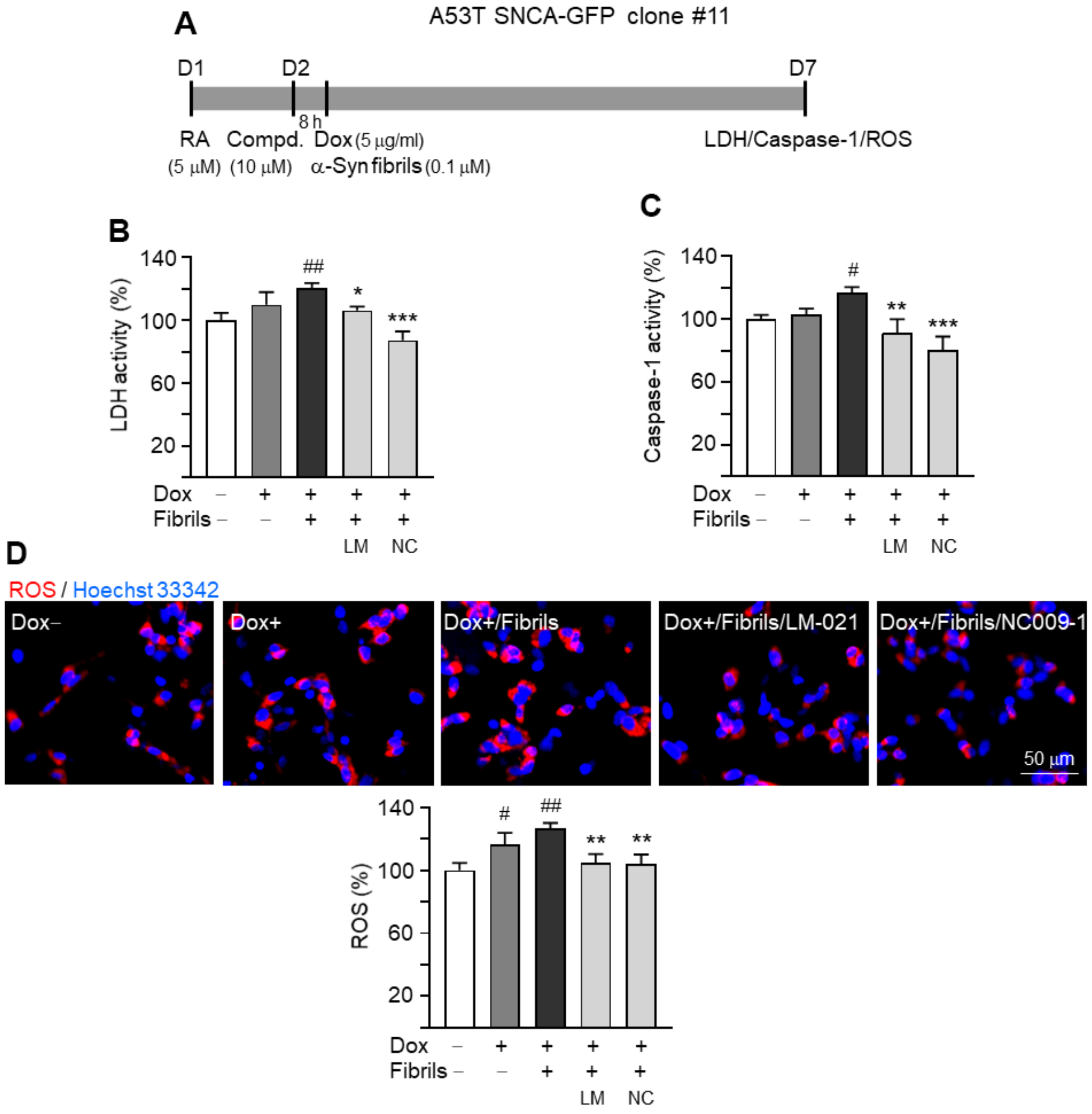

Supplementary Figure 3. Neuroprotective effects of LM-021 and NC009-1 on A53T-11 SNCA-GFP BE(2)-M17 cells. (**A**) Experimental flow chart. Cells were seeded in the presence of retinoic acid (RA, 5 μM) on day 1. On day 2, LM-021 or NC009-1 (10 μM) was added to cells for 8 h, followed by induction of SNCA-GFP expression (Dox, 5 μg/ml) and addition of α-synuclein fibril (0.1 μM). On day 7, LDH release, caspase-1 activity and ROS were assessed. (**B**) LDH release, (**C**) caspase-1 activity, and (**D**) ROS production of A53T-11 SNCA-GFP BE(2)-M17 cells with or without induced SNCA-GFP expression, preformed fibril addition, or LM-021/NC009-1 treatment (n = 3). *P* values: comparisons between with and without doxycycline addition (#: $P < 0.05$, ##: $P < 0.01$), or between with and without compound treatment (*: $P < 0.05$, **: $P < 0.01$, ***: $P < 0.001$).

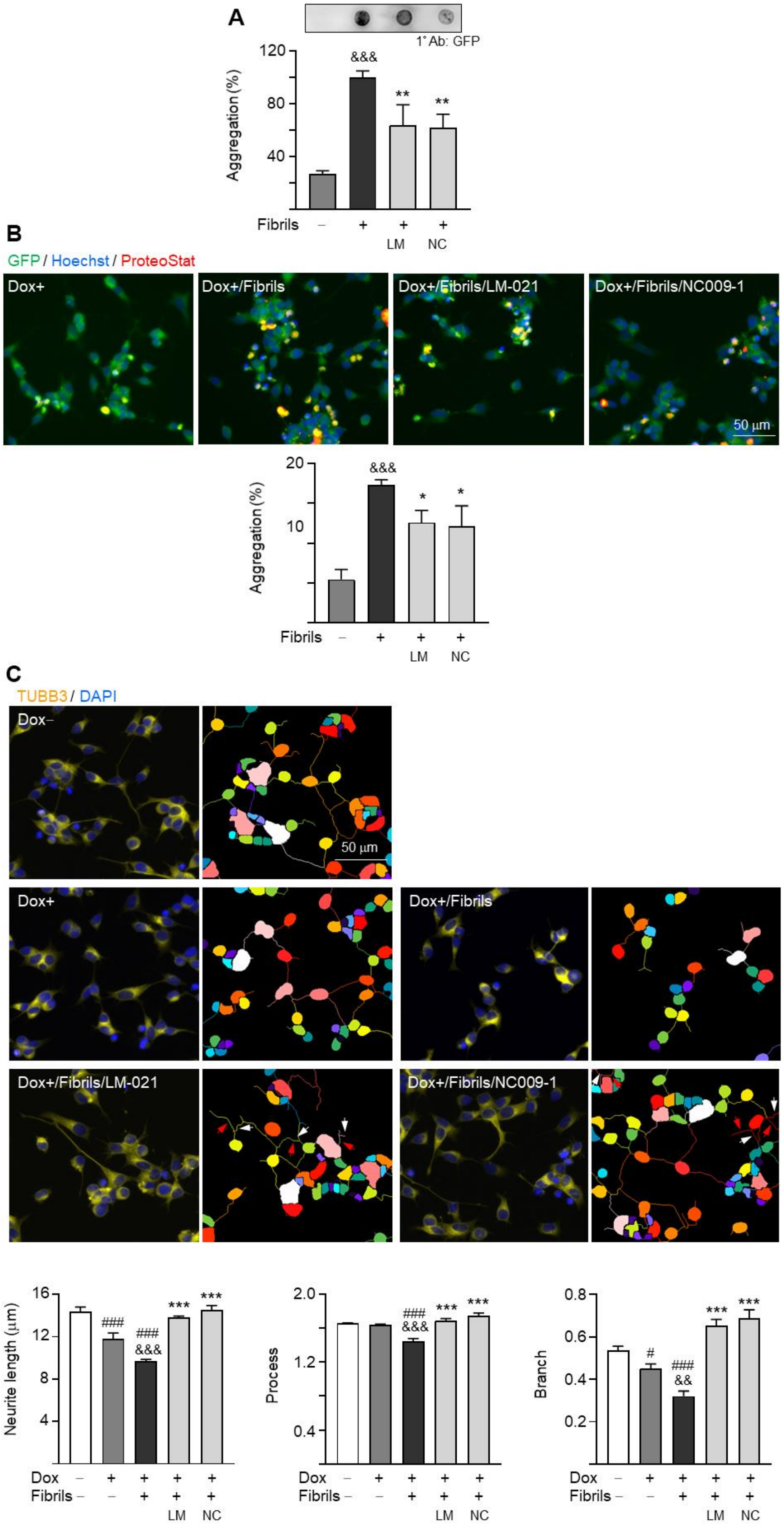

Supplementary Figure 4. α-Synuclein aggregation reduction and neurite outgrowth promotion of LM-021 and NC009-1 on A53T-11 SNCA-GFP BE(2)-M17 cells. As described, retinoic acid was added to the cells on day 1, and compound, doxycycline and

α-synuclein fibril were added on day 2. On day 7, α-synuclein aggregation and neurite outgrowth were assessed. (**A**) α-Synuclein aggregates measured by filter trap assay using a GFP antibody. To normalize, the relative α-synuclein aggregate with fibril addition was set as 100%. (**B**) Fluorescent microscopy images of A53T-11 SNCA-GFP-expressing cells (green) with or without preformed fibril addition or compound treatment. Nuclei were detected with Hoechst 33342 (blue) and overlapped SNCA-GFP and ProteoStat®-stained (red) aggregates marked (yellow). Quantification of percentage of aggregated cells was shown below (n = 3). (**C**) Fluorescent microscopy images of A53T-11 SNCA-GFP cells stained with TUBB3 for neurite outgrowth quantification with or without preformed fibril addition or compound treatment, with nuclei detected (DAPI, blue). Also shown were images of the neurites and cell bodies being outlined for neurite outgrowth quantification. In cells with NC009-1 treatment, processes and branches are indicated with red and white arrows, respectively. Quantification of neurite length, brunch and process was shown below (n = 3). *P* values: comparisons between with and without doxycycline addition ($^{\#}$: $P < 0.05$, $^{\#\#\#}$: $P < 0.001$), with and without fibrils addition ($^{\&\&}$: $P < 0.01$, $^{\&\&\&}$: $P < 0.001$), or between with and without compound treatment (*: $P < 0.05$, **: $P < 0.01$, ***: $P < 0.001$).
